# Multi-epitope vaccine targeting SARS-CoV-2 omicron S and N proteins promotes enhanced immunity: a computational approach

**DOI:** 10.3389/fimmu.2026.1906485

**Published:** 2026-09-08

**Authors:** Xinyi Xu, Arslan Habib, Naishuo Zhu

**Affiliations:** 1 Laboratory of Molecular Immunology, State Key Laboratory of Genetic Engineering, School of Life Sciences, Fudan University, Shanghai, China; 2 Department of General Surgery, Xinhua Hospital Affiliated to Shanghai Jiao Tong University School of Medicine, Shanghai, China; 3 Shanghai Key Laboratory of Biliary Tract Disease Research, Shanghai, China; 4 Yaoyanda Technology Development Co., Ltd., Suzhou, Jiangsu, China

**Keywords:** Gb-1, immune simulation, immunoinformatics, molecular docking, molecular dynamics simulation, multi-epitope vaccine, SARS-CoV-2

## Abstract

**Background:**

The emergence of severe acute respiratory syndrome coronavirus 2 (SARS-CoV-2) led to the COVID-19 pandemic, which resulted in millions of deaths globally and had profound social, economic, and political consequences. Although effective vaccines and antiviral therapies have substantially reduced the global burden of COVID-19, the continued emergence of viral variants highlights the need for next-generation effective vaccine strategies capable of providing broader and more durable immune response.

**Methods:**

In this work, we provide an immunoinformatic approach for multi-epitope vaccine (MEV) design and prediction. Based on the spike (S) and nucleocapsid (N) proteins of SARS-CoV-2, immunoinformatic methods were used to identify the epitopes for B cells, cytotoxic T lymphocytes (CTL), and helper T lymphocytes (HTL). The B cell, CTL, and HTL epitopes were conjugated with flexible linkers GSG, GSGG, and a Gb-1 peptide conjugated to the C-terminal of the MEV ccandidate.

**Results:**

The final MEV candidate exhibited favorable predicted characteristics, with a molecular weight of approximately 55.47 kDa and a length of 498 amino acid residues. Computational analyses indicated that the designed construct was antigenic, non-toxic, non-allergenic, and possessed suitable physicochemical properties and predicted solubility, supporting its potential as a vaccine candidate for further investigation. Molecular docking analysis demonstrated favorable interactions between the MEV construct and selected Toll-like receptors (TLRs), while molecular dynamics (MD) simulations suggested the stability of the vaccine-receptor complexes throughout the simulation period. Furthermore, C-ImmSim-based immune simulation predicted the induction of both humoral and cellular immune responses following the proposed immunization schedule. Collectively, these findings highlight the potential of the designed MEV construct as a computationally optimized vaccine candidate and provide a framework for future experimental evaluation.

**Conclusion:**

This study presents a computationally designed MEV candidate against SARS-CoV-2 by integrating immunoinformatics approaches, structural modeling, molecular docking, molecular dynamics simulations, and immune response prediction. The findings suggest that the proposed MEV construct may possess favorable immunogenic and structural properties; however, experimental validation through *in vitro* and *in vivo* studies remains essential to confirm its safety, immunogenicity, and protective efficacy. The proposed approach provides a valuable strategy for accelerating rational vaccine design and may serve as a foundation for future development of experimentally validated vaccine candidates.

## Introduction

Over the past three years, a persistent global coronavirus disease 2019 (COVID-19) pandemic has been caused by SARS-CoV-2 and several emerging variants or subvariants. The worldwide epidemic has been particularly devastating to elderly patients with underlying diseases and has resulted in over 6 million COVID-19-related deaths (1). The clinical manifestations of COVID-19 vary considerably among individuals; some individuals remain asymptomatic or experience mild symptoms, whereas others develop severe respiratory failure or even succumb to the disease. The underlying mechanisms responsible for this marked variation in disease severity remain incompletely understood (2). Coronaviruses (CoVs) are classified into four major genera: Alphacoronavirus (α-CoV), Betacoronavirus (β-CoV), Gammacoronavirus (γ-CoV), and Deltacoronavirus (δ-CoV). These viruses infect a diverse range of hosts, including birds and mammals, and are named after the crown-like appearance of their surface spike proteins (3). SARS-CoV-2 is a β-coronavirus belonging to the family Coronaviridae, order Nidovirales, and subfamily Orthocoronavirinae. Its nucleocapsid exhibits helical symmetry (4). As a positive-sense, single-stranded RNA virus, the SARS-CoV-2 genome codes for several key components. These include vital structural proteins like the spike (S), envelope (E), membrane (M), and nucleocapsid (N), alongside viral replicases, polyprotein-cleaving proteases, and various uncharacterized proteins (5). Within the SARS-CoV-2 genome, 14 open reading frames (ORFs) including ORF1a and ORF1ab encode the virus’s essential machinery. This genetic blueprint produces 16 non-structural proteins (NSP1-16) alongside four critical structural components: the S glycoprotein, E, M, and N proteins (6).

In contrast to some other betacoronaviruses, SARS-CoV and SARS-CoV-2 do not encode a hemagglutinin esterase (HE) protein (7). SARS-CoV-2 infects host cells by binding to the angiotensin-converting enzyme 2 (ACE2) receptor through its S protein, which consists of the S1 and S2 subunits. The receptor-binding domain (RBD) within the S1 subunit mediates the attachment of SARS-CoV-2 to the angiotensin-converting enzyme 2 (ACE2) receptor, whereas the S2 subunit facilitates membrane fusion between the virus and host cell. Following membrane fusion, the viral genome is released into the host cell cytoplasm, initiating the viral replication cycle (8). Walls et al.’s study examined the structure, function, and immunogenicity of the multifunctional S protein (9). Anti-CoV treatments primarily aim to prevent the virus from interacting with receptors and to halt its replication (10, 11). Immunization induces the host immune system to produce antibodies that target RBD of S protein, thereby blocking its interaction with the ACE2 receptor. Therefore, immunization against the RBD would be crucial in preventing the virus from attaching itself to the host cell, thereby limiting viral invasion and infection. As a result, the RBD is an important target when developing anti-CoV treatments (8). The RBD shares 83% sequence similarity and 75% structural commonality with SARS-CoV. Therefore, it has been revealed that the residues in the receptor-binding motif (RBM) in the RBD are 59% identical (12). The prevalence of COVID-19 has been largely connected to the replacement of key residues in the RBD of SARS-CoV-2. Furthermore, distinct antigenicity and antibodies against the RBD that are ineffective against the other have been produced by the sequence difference in RBM between SARS-CoV and SARS-CoV-2 (9). Therefore, developing antigenic vaccines targeting the SARS-CoV-2 S protein is crucial, as these vaccines can induce immune responses, including neutralizing antibodies and T-cell activation, that interfere with spike protein-ACE2 receptor interactions and limit viral entry into host cells (13).

During the viral lifecycle, the N protein drives virion assembly by packaging the genomic RNA and engaging directly with membrane-bound proteins (14). Since it is highly stable across variants compared to the S protein, this strongly immunogenic component offers a compelling alternative target for multi-antigen vaccine platforms (15). Although the N protein has been reported to be expressed in association with infected cells, its protective immunological role is thought to arise primarily from the induction of antigen-specific T-cell responses and the activation of innate immune mechanisms that facilitate the recognition and elimination of infected cells (16). Unlike the S protein, the N protein is not exposed on the surface of the virion. Nevertheless, numerous preclinical studies have evaluated N-based vaccine candidates, investigating the N protein both as a standalone immunogen and in combination with the S protein to enhance protective immune responses (17, 18). A recent preclinical study demonstrated that immunization with both the N and S proteins provided enhanced protection against viral challenge and reduced viral dissemination to the brain in mice (19). However, the inclusion of the N antigen is not without potential limitations. Early studies on SARS-CoV suggested that nucleocapsid-specific immune responses might contribute to immunopathology following subsequent viral exposure under certain experimental conditions (20). Furthermore, although the N protein is generally more conserved than the S protein across coronaviruses, it remains susceptible to adaptive mutations. Nevertheless, incorporating T-cell-targeted vaccine strategies remains advantageous because mutations affecting a specific T-cell epitope are unlikely to compromise immune protection across individuals with diverse major histocompatibility complex (MHC) haplotypes (21).

Several innovative strategies have been developed in recent years to reduce the time and cost associated with vaccine development. Among these approaches, reverse vaccinology integrates immunogenomic analysis with bioinformatics tools to identify potential vaccine targets and facilitate the rational design of novel vaccine candidates (22). Unlike conventional vaccine development approaches, this strategy reduces both the cost and time required for vaccine development. Furthermore, it minimizes the number of experimental steps needed for the initial identification and optimization of potential vaccine candidates (23). The traditional method of developing vaccines, which uses entire organisms or proteins, increases the risk of adverse reactions and produces an enormous antigenic load. Engineering an MEV offers a direct solution to these challenges. By combining short immunogenic peptides with targeted adjuvants and optimized structural linkers, an MEV can spark a highly focused immune defense while minimizing the risk of allergenic side effects. Compared to single-epitope designs, MEVs have the potential to provide broader target specificity and may exhibit favorable predicted structural stability while enabling the incorporation of multiple epitopes into a single MEV candidate. However, these potential advantages require experimental confirmation (24). Additionally, by incorporating both T cell and B cell epitopes, the MEV construct is predicted to elicit both humoral and cellular immune responses.

Adjuvants are commonly used to enhance antigen-specific immune responses and may reduce antigen requirements. Therefore, their inclusion in MEV candidate is intended to improve the predicted immunogenic potential of the construct (25). Although the global burden of COVID-19 has been substantially reduced through the widespread deployment of effective vaccines and the availability of antiviral therapies, SARS-CoV-2 continues to evolve, giving rise to variants with enhanced transmissibility and varying degrees of immune escape. Waning vaccine-induced immunity and the continuous emergence of novel viral variants highlight the need for next-generation vaccine strategies capable of eliciting broader and more durable immune responses. In this context, computational immunoinformatics provides a powerful platform for the rational design of MEVs targeting conserved and immunologically relevant regions of viral proteins, thereby supporting the development of improved vaccine candidates for future SARS-CoV-2 variants.

The development of a SARS-CoV-2 vaccine has advanced significantly, but there are still a number of obstacles to overcome, such as an ongoing emergence of viral variations, alterations inside immunodominant domains, declining immunity, and the requirement for vaccine platforms that may elicit broader immune protection. In order to get around some of the drawbacks of traditional single-antigen techniques, more recent research has delved into next-generation vaccine strategies, such as MEV platforms. Fewer research have examined integrated designs including conserved epitopes from different viral proteins, such as S and N proteins, along with appropriate immune-enhancing adjuvants, whereas many reported computational vaccine candidates concentrate solely on S-derived epitopes (25). Additionally, in order to improve the selection and optimization of possible vaccine candidates, a thorough computational evaluation that incorporates epitope prediction, population coverage analysis, structural characterization, molecular docking, MD simulation, and immune response prediction is still necessary. Therefore, more research is needed to develop rationally constructed MEV models that might offer more comprehensive and long-lasting immune responses against newly emerging SARS-CoV-2 variants.

**Figure 1:**
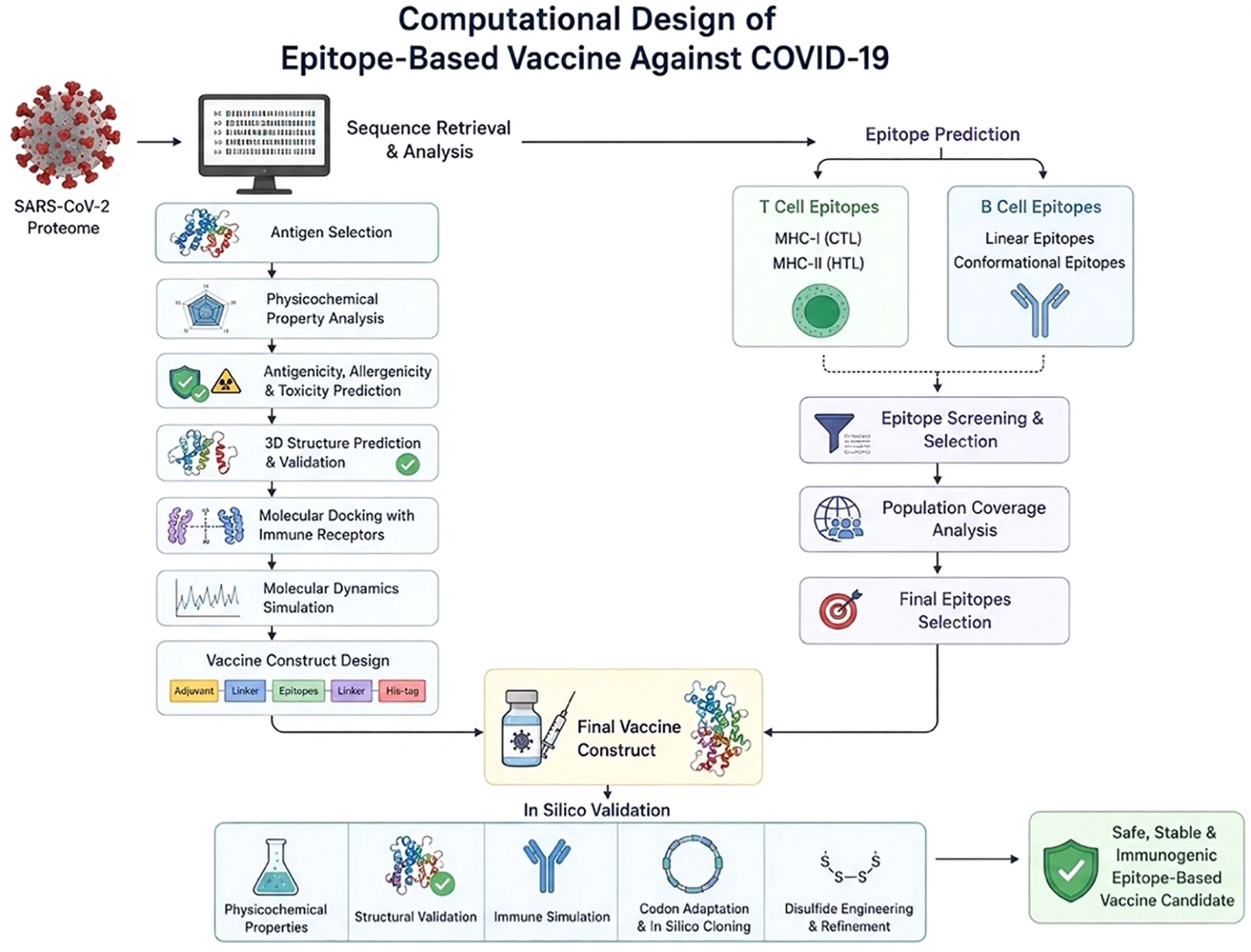
Schematic workflow outlining the computational pipeline used to design and evaluate the MEV candidate. The BioRender tool was used to generate a computational design image.

For specific vaccine development, immunoinformatic strategies are currently validated alternatives that can be used to all SARS-CoV-2 variants. Therefore, we used immunoinformatic techniques in this investigation to predict MEVs against the S and N proteins of the Omicron variant in conjugation with Gb-1 (**Figure 1**). B cell and T cell epitopes with MHC- I/II alleles were predicted using the SARS-CoV-2 Omicron S protein after it was examined for antigenicity. The expected epitopes’ antigenicity, and worldwide coverage were examined. The structural consistency, physiochemical characteristics, immunogenicity, toxicity, and antigenicity of the produced MEVs were also validated using a variety of immunoinformatic techniques. To computationally assess the predicted structural stability and binding dynamics of the designed MEV in complex with human immune receptors, molecular docking followed by MD simulations was performed. Subsequently, *in silico* codon optimization and cloning were performed to improve the predicted expression efficiency of the MEV construct by adapting its codon usage for expression in *Escherichia coli (E. coli)*. The final vaccine construct incorporated appropriate molecular linkers and the Gb-1 adjuvant to facilitate epitope presentation and enhance the predicted immune response. Collectively, these computational findings provide a foundation for the rational design of vaccine candidates targeting the SARS-CoV-2 Omicron variant. However, comprehensive *in vitro* and *in vivo* studies are required to validate the expression, immunogenicity, safety, and protective efficacy of the proposed vaccine construct.

## Materials and methods

### Retrieval of omicron protein sequences and multiple alignment

Complete SARS-CoV-2 Omicron spike (S) and nucleocapsid (N) protein sequences were retrieved from the NCBI database. More than 100 complete S and N protein sequences were collected and used for subsequent sequence analysis. The amino acid sequences were downloaded in FASTA format, and the representative GenBank accession numbers WAW22208.1 (S protein) and WAW22215.1 (N protein) are provided as reference sequences. To identify conserved regions and sequence variations among the retrieved Omicron isolates, multiple sequence alignment was performed using Clustal Omega (ClustalO) and the STRAP program. The aligned sequences were subsequently analyzed using the MUSCLE web server to generate consensus sequences, which were used for all downstream epitope prediction and vaccine design analyses.

### B cell epitope prediction

B cell epitopes are essential components of epitope-based vaccines because they stimulate humoral immune responses through the production of antigen-specific antibodies. An ideal peptide vaccine should induce durable humoral immunity, providing long-lasting antibody-mediated protection similar to that achieved following effective vaccination or natural infection. Therefore, the identification of immunogenic B cell epitopes is a critical step in the rational design of peptide-based vaccines. Linear B-cell epitopes within the SARS-CoV-2 S sequence were identified using two complementary prediction methods: BepiPred-2.0 and the IEDB BepiPred Linear Epitope Prediction tool. Both analyses were performed using the default prediction parameters and threshold values recommended by the respective servers. BepiPred-2.0 employs a random forest machine-learning algorithm trained on experimentally resolved antibody-antigen complex structures to predict linear B-cell epitopes based on amino acid sequence and structural features. The predicted epitopes obtained from both methods were subsequently compared and selected for further analysis according to the predefined epitope-screening criteria described in this study. This approach bridges structural data with a vast library of linear epitopes harvested from the IEDB database. To further validate the predicted linear B-cell epitopes, iBCE-EL, an ensemble learning-based prediction framework, was employed as an independent screening approach. The tool evaluates candidate peptides based on sequence-derived and physicochemical features, including amino acid composition, dipeptide characteristics, and other relevant properties. iBCE-EL integrates multiple machine-learning classifiers to assign each peptide a predicted epitope classification along with a probability score. Peptides showing favorable prediction outcomes were considered for subsequent vaccine design analyses (26, 27). To evaluate conformational B cell epitopes, we analyzed the spatial and geometrical properties of the target proteins using the ElliPro server. This method evaluates three-dimensional antigen architecture and represents one of the most reliable structure-based prediction tools available, historically outperforming alternative modeling techniques with a peak Area Under the Curve (AUC) value of 0.732.

### CTL epitope prediction and immunogenicity assessment

In order to develop a unique antigenic vaccine candidate against the novel strain, highly immunogenic and antigenic epitopes were derived from the sequences of the S and N proteins. The IEDB and NetMHC 4.0 web services were used to predict binding epitopes to MHC I alleles. A peptide length of 8–12 amino acids and an IC_50_ value of less than 200 nM were among the criteria used to identify MHC-I-binding alleles. Potential MHC class I-binding epitopes were identified using peptide lengths of 8–12 amino acids and an IC_50_ threshold of <200 nM. Because lower IC_50_ values indicate stronger predicted binding affinity, peptide-MHC interactions were classified as high (≤50 nM), intermediate (50–500 nM), or low (500–5000 nM) affinity. The selected peptide candidates were subsequently evaluated using the IEDB MHC class I immunogenicity prediction tool to estimate their potential to elicit CD8^+^ T cell immune responses. Unless otherwise specified, all analyses were performed using the tool’s default parameters, and the highest-ranked predicted immunogenic epitopes were selected for further investigation.

### HTL epitope and HLA CD4^+^ immunogenicity prediction

The IEDB server produced 15mer HTL epitopes that demonstrated a strong affinity for human MHC II molecules. The HTL epitopes were sorted by the IC_50_ score, and those with an IC_50_ value less than 50 nM were regarded as good binders. To identify HTL epitopes, we used the combinatorial method suggested by the IEDB. This approach combined the methods of NN-align, SMM-align, CombLib, Sturniolo, and NetMHCIIpan. A lower %age rank would indicate a higher binding affinity, the ability to release IFN-γ, and the capacity to develop imperative features. Because the percentile rank is inversely related to the predicted binding affinity, lower percentile ranks indicate stronger peptide-MHC binding. The IEDB Analysis Resource was used to predict the CD4 immunogenicity of the selected proteins. Without requiring HLA typing, the CD4episcore predicts the immunogenicity of antigens for CD4^+^ T cells at the demographic level. Its effectiveness has been confirmed in several contexts, such as distinct antigen sources, ethnic groups, and methods for identifying epitopes.

### Highly promising epitopes selection

The selection thresholds for epitope screening were established based on previously reported studies and experimentally validated epitope datasets. The screening strategy was designed to maximize the identification of high-quality candidate epitopes while maintaining appropriate specificity. For each epitope category, predefined criteria were applied, including predicted immunogenicity, antigenicity, HLA-binding affinity, conservation, and other relevant physicochemical properties. The dataset primarily consisted of immunogenic and non-immunogenic epitopes derived from the Omicron variant S and N proteins, which were compiled through a meta-analysis of multiple studies focusing on MHC class I and MHC class II epitopes. Following sequential screening steps, only epitopes meeting the established selection criteria were retained for subsequent analyses and incorporation into the final multi-epitope vaccine construct.

### Proteasomal cleavage/TAP transport

Proteasomal cleavage and transporter-associated antigen processing (TAP) were conducted using NetCTL 1.2. The website employs an algorithm that forecasts the effectiveness of proteasomal C-terminal cleavage, MHC-I binding, and TAP transport. TAP transport potential was assigned a weight of 0.05 and C-terminal cleavage a weight of 0.15 under the default conditions (28).

### Evaluation of IFN-γ inducing epitopes

IFN-γ plays a critical role in controlling viral replication. This potent cytokine coordinates both native and antigen-specific immune branches, driving the activation of natural killer (NK) cells and macrophages. Moreover, IFN-γ increases MHC’s reaction to antigens. IFN-γ epitopes were identified using the IFN-γ epitope server. The low-scoring HTL epitopes were added to the IFN-γ epitope server. The support vector machine (SVM) hybrid technique was used to predict positive IFN-γ induction. We selected the final HTL epitopes based on a dual-screening framework: robust MHC-II binding affinities and strong IFN-γ induction profiles, both of which work synergistically to stimulate helper T cells (29).

### Population coverage estimation

Population coverage assessments leverage known HLA allele frequencies to forecast a vaccine’s geographical and demographic reach. We calculated these metrics by cross-referencing the selected T cell epitopes of S and N proteins with their corresponding HLA binding restrictions. The IEDB Population Coverage tool was used to estimate the global population coverage of the selected epitopes based on their corresponding HLA allele associations.

### Prediction of antigenicity, allergenicity, and solubility analysis

VaxiJen 2.0 and ANTIGENpro tools were used to forecast the antigenic potential of model vaccine components using a preset threshold value of >0.5. Through the auto cross-covariance (ACC) transformation, VaxiJen converts raw protein sequences into uniform vectors that represent fundamental physicochemical amino acid properties. Conversely, AntigenPro is a sequence-based predictor of protein antigenicity that is independent of pathogens (30, 31). The allergenicity impacts of vaccine candidate were examined using the AllergenFP 1.0 and AllerTOP 2.0 tools for allergenicity analysis, which have an accuracy of 85% and a cut-off of -0.4. Vaccines with scores below a certain level are regarded as non-allergenic (32). The solubility characteristics of the vaccine model that will facilitate the efficient production of the vaccine design in *E. coli* plasmid were determined using the SOLpro program (33).

### Physicochemical properties and toxicity analysis

Evaluation of the physicochemical characteristics is an important step in the computational assessment of engineered vaccine candidate, providing predictive insights into their stability, solubility, and suitability for further experimental development. Accordingly, we evaluated parameters such as molecular weight, theoretical isoelectric point (pI), instability index, aliphatic index, estimated half-life, and grand average of hydropathy (GRAVY) using the ExPASy ProtParam server (34). Furthermore, to screen out potentially toxic sequences, the toxicity of the finalized MHC-I, MHC-II, and B cell epitopes was calculated via the ToxinPred platform, which leverages an SVM architecture to classify peptides as either toxic or non-toxic (35).

### Epitopes hydropathy assessment

All epitopes selected for inclusion in the final MEV construct were evaluated for their hydrophilicity, as surface-accessible and hydrophilic epitopes are generally more likely to be recognized by the host immune system. Therefore, hydrophilicity was considered an important criterion during epitope selection to enhance the predicted immunogenic potential of the vaccine construct. The ProtParam tool was used to analyze the GRAVY score in order to assess the hydrophobicity of the selected epitopes. Mathematically, the GRAVY score represents the sum of all hydropathy values within a protein sequence divided by its total residue count. The sign of the resulting value dictates the protein’s overall solubility behavior: a positive score denotes a predominantly hydrophobic nature, while a negative value signifies overall hydrophilicity.

### MHC-restricted alleles cluster analysis

MHC-restricted epitope prediction was performed using IEDB MHC-I and MHC-II binding prediction tools. The IEDB reference allele set was selected to maximize global population representation. The predicted peptide–HLA interactions were subsequently evaluated using the MHCcluster v2.0 server, which groups HLA molecules according to similarities in their peptide-binding specificities and visualizes functional relationships through a static heat map (36).

### MEV structure and modeling

Candidate epitopes were rigorously prioritized based on a stringent multi-tier filter: optimal antigenicity, a complete absence of allergenic or toxic motifs, lack of alignment with the host human proteome, and high sequence conservation across target strains. Only the HTL epitopes capable of inducing cytokines were considered for vaccine development. During the development of MEV construct, highly immunogenic epitopes that satisfied the predefined selection criteria after multiple rounds of computational screening were selected for incorporation into the final vaccine design. To enhance the immunological potential of the proposed MEV candidate, a GP-2 binding peptide (Gb-1) identified previously in our laboratory through phage display screening was incorporated into the design. Preliminary experimental characterization of Gb-1 demonstrated its immunostimulatory properties and its ability to preferentially promote a Th2-type immune response. Based on these observations, Gb-1 was included as an immunomodulatory ligand with the aim of facilitating immune recognition and potentially improving antigen delivery. To preserve the integrity of the selected B-cell, CTL, and HTL epitopes while maintaining accessibility of the ligand, Gb-1 was conjugated to the C-terminus of the multi-epitope MEV candidate using appropriate linker sequences. The potential contribution of Gb-1 to the immunogenic properties of MEV candidate should be regarded as a design hypothesis based on prior experimental observations and requires further experimental validation in the context of the present vaccine candidate (37). To construct the MEV, an adjuvant was incorporated based on its reported immunostimulatory properties and its potential to enhance the predicted immunogenicity of MEV candidate. A flexible GSGG linker was used to connect the adjuvant to the downstream epitope regions with the aim of preserving the structural independence of the individual components. In addition, the Gb-1 (GP-2 binding) peptide was incorporated into the construct based on its previously reported immunostimulatory characteristics and its potential to facilitate immune recognition. Finally, GSG and GSGG linkers were strategically employed to join the selected B-cell, CTL, and HTL epitopes into a continuous linear polypeptide while minimizing potential structural interference between adjacent epitopes (38).

### Evaluation of physicochemical parameters of MEV candidate

Higher antigenicity is an important aspect of vaccine development because it promise that the host immune system will recognize the antigen, stimulating immune cells and provoke an immunological response (39). Computational evaluation of potential allergenicity represents an important safety assessment, providing predictive evidence of the likelihood that the MEV candidate may elicit allergenic or hypersensitivity reactions and informing subsequent experimental safety evaluation. Concurrently, profiling the molecule’s diverse physicochemical parameters is vital to guarantee both clinical safety and optimal therapeutic performance at computational level. The predicted antigenicity of the designed MEV candidate was evaluated using VaxiJen v2.0 with a threshold value of 0.4. The potential allergenicity of the construct was assessed using AlgPred and AllerTop v2.0 servers. The final MEV candidate physicochemical properties, such as the pI value, half-life, and GRAVY value, were again evaluated using ProtParam. The solubility profile of the finalized vaccine was calculated via the SOLpro tool hosted on the SCRATCH platform. Maintaining adequate solubility is a fundamental prerequisite for successful formulation development, as it prevents aggregation and ensures effective delivery inside the host cell.

### MEV secondary and tertiary structure prediction

PSIPRED program was used to analyze the 2D structure of protein while keeping all of the default settings. PSIPRED was set up to evaluate biomolecules’ secondary structure, transmembrane topology, helices, folds, and domain recognition (40). To further validate the results, 2D structural evaluation was performed by SPOMA and Phyre2 tools for correlation and additional confirmation. The three-dimensional (3D) structure of the MEV candidate was predicted using the RoseTTAFold online server (BOINC version 7.6.22) without relying on closely related homologous templates. RoseTTAFold is a neural network-based technique that uses a “three-track” strategy. It takes into account possible 3D structures, interactions between amino acids inside proteins, and trends in protein sequences. This integrated framework seamlessly bridges a protein’s primary amino acid sequence, chemical constituents, and tertiary folded structure into a unified predictive model. Leveraging this holistic architecture, RoseTTAFold achieves exceptional structural accuracy that directly rivals DeepMind’s AlphaFold pipeline (41).

### MEV candidate 3D refinement and validation

Experimental validation is essential for confirming the accuracy and biological relevance of computationally predicted structures in biomedical applications, as 3D modeling alone may not fully represent the native conformation of a protein. Therefore, 3D structure refinement was performed to improve the predicted structural quality by optimizing the initial model, correcting potential local structural irregularities, and maintaining the overall architecture of the vaccine construct. The MEV candidate structure was refined using the GalaxyRefine tool. This server uses a CASP10-tested method for dynamics simulation and refinement, leading to improved structures (42). The Ramachandran plot server was employed for model verification in order to identify potential structural problems. The main-chain conformational inclinations of an amino acid are shown graphically by a Ramachandran plot (43). Additionally, the ProSA-web tool was used, which uses a Z-score to represent the overall quality of the protein models. Z-scores that fall outside of the typical range are regarded as faulty structures (44).

### MEV candidate disulfide engineering analysis

Leveraging targeted molecular interactions to maximize antigen stability represents a vital phase in vaccine development, directly dictating downstream immunogenicity and formulation shelf-life. The disulfide design of the vaccine protein was examined using Disulfide by Design 2 v12.2. The aim of this server is to locate potential sites where disulfide connections are more likely to occur in a protein structure. The program makes use of computational techniques to predict protein structure. By utilizing a geometric coordinate framework derived from native disulfide bonds, this approach mathematically derives the *X*3 torsion angle directly from the fifth Cbeta-Cbeta interatomic distance (45).

### Molecular docking of MEV candidate with TLRs

To evaluate the potential of the designed vaccine construct to elicit immune responses, the interaction between the final MEV candidate and its selected immune receptors was investigated. The primary function of TLRs in initiating innate immune responses and bridging innate and adaptive immunity led to their selection for docking study. Because of their proven capacity to identify microbial proteins and protein-associated molecular patterns, TLR2 and TLR4 were chosen as biologically relevant targets for assessing possible interactions with a protein-based multi-epitope vaccine design. Furthermore, TLR3, TLR7, and TLR8 were added as exploratory computational targets to look at possible molecular interactions with receptors involved in antiviral immune signaling. These receptors are mostly engaged in sensing viral nucleic acids within endosomal compartments. Instead of assuming direct receptor activation or physiological recognition of MEV candidate, the docking analyses were designed to evaluate expected binding compatibility.

A valuable method for calculating binding energies and evaluating interactions between epitopes and HLA molecules is the molecular docking method. Following multi-parameter optimization, the finalized MEV candidate was subjected to molecular docking against the ligand-binding cavities of six distinct TLRs. Specifically, we targeted TLR-2, 3, 4, 5, 7, and 8, using structural coordinates obtained from the RCSB Protein Data Bank under PDB IDs 6NIG, 5GS0, 4G8A, 3J0A, 5GMG, and 4QC0, respectively. Additionally, we used the ClusPro web server to dock the MEV candidate with TLRs (46). ClusPro performs rigid-body docking using the rapid Fourier transform correlation method. The platform subsequently decomposes the total potential energy into its individual thermodynamic components: hydrogen bonding, atomic contact, and both attractive and repulsive van der Waals forces. PyMOL v2.3.4 software was used to improve the 3D architectures of both TLRs and the SARS-CoV-2 MEV candidate for energy. To ensure robust prediction accuracy, protein-protein docking was performed across multiple online servers using water-depleted PDB coordinates for the MEV and TLR models. Structural geometry and spatial orientations were visualized during the docking analysis using the UCSF Chimera v1.13.1 suite.

In the second stage of docking, the clusters of docked complexes were organized using ClusPro 2.0, occupying the center and lowest energy ratings. Here, the energy score was determined using the ClusPro program and the equation E = 0.40Erep + 20.40Eatt + 600Eelec + 1.00EDARS (46). Lower binding energy thresholds were used to infer stronger affinity profiles. Post-docking, the binding free energies of the vaccine-TLR complexes were computed via MM-GBSA under default parameters, and the structural dynamics were validated through MD simulations. The resulting complex geometries were visualized using Discovery Studio Visualizer. Finally, we thoroughly interrogated the interfacial protein-protein boundaries using PDBsum and profiled the stabilizing H-bonds using the LigPlot^+^ tool (47).

### MD Simulation of MEV-receptor complex

The biophysical flexibility of the finalized vaccine-TLR4 complex was evaluated through normal mode analysis (NMA) using the computational tools available on the iMODS platform. The analysis assessed structural flexibility parameters, including deformability, covariance, predicted B-factors, and eigenvalues, to characterize the dynamic behavior and stability of the protein complex. Such analyses provide computational insights into the predicted structural stability and conformational dynamics of the protein (48).

### Codon optimization and in silico cloning of MEV

Codon optimization is required in accordance with the particular host organism for the production of a foreign gene. Codon optimization was performed to maximize the expression potential of the vaccine candidate in an *E. coli* K-12 expression system. Utilizing the JCAT platform, the polypeptide sequence was back-translated into a nucleotide sequence engineered to filter out specific structural liabilities: restriction endonuclease sites, internal ribosome entry sites, and rho-independent terminators. We utilized the calculated Codon Adaptation Index (CAI) and overall GC percentage to evaluate expression fitness. To complete the *in silico* cloning workflow, the finalized gene was integrated into a pET-28a(+) plasmid vector map using the SnapGene (49, 50).

### Immune simulations of MEV candidate

The C-ImmSim simulation tool was used to predict the immune feedback profile and vaccine immune simulation. It employed an agent-based model and machine learning techniques to assess immunological interactions using a position-specific scoring matrix (PSSM). Two injections of the MEV preventive Omicron variant vaccine were given, and the simulation steps domain was changed to 1050 to account for the four-week interval between the first and second immunization doses using the default simulation parameters, which did not include any LPS or volume. Each time step was set at 1 and 84, which corresponded to eight hours in real time. Throughout the trial, other settings stayed at their predetermined values.

## Results

### Sequences retrieval and epitopes screening

The continuous emergence of novel SARS-CoV-2 variants globally presents a critical challenge to ongoing public health interventions. These evolving lineages display pronounced genetic modifications when contrasted with the ancestral wild-type strain isolated in Wuhan, China, in late 2019. Additionally, the S protein mutations may worsen the severity and infectivity of SARS-CoV-2 and perhaps reduce the efficacy of vaccinee. In order to aid in the development of a vaccination against the novel variant, computational modeling and integrated immunoinformatics were employed in the current study to develop a MEV. In total, we extracted over 100 complete S and N protein sequences. We generated a baseline consensus sequence by cross-aligning the candidate strains using several alignment algorithms. To determine whether this core sequence could trigger a protective immune response, we profiled its baseline antigenicity via the VaxiJen v2.0 platform. The TMHMM v2.0 server was employed to assess the transmembrane helices of proteins, and the results revealed that the number varied between 0 and 1. We used the AllergenFP v1.0 program, which is widely used in this type of evaluation, to evaluate the allergic response from protein sequences. To assess the MEV candidate antigenicity and other physicochemical properties, we calculated a number of metrics, including GRAVY, theoretical pI, and half-life scores. Finally, we chose a S and N protein with the highest antigenic score out of all the proteins that were evaluated based on the antigenicity. Downstream epitope prediction algorithms were deployed on the consensus sequence to identify potential immunogenic peptide candidates. All of the computational tools utilized in this experiment are listed in **Table 1**.

**Table 1:** Several immunoinformatic tools to guide the formulation and development of the vaccine.

Parameter	Server name	Server link
Consensus sequence	muscle server	https://www.ebi.ac.uk/Tools/msa/muscle/
B cell prediction	IEDB	http://tools.iedb.org/bcell/
B cell prediction	BepiPred-2.0	http://www.cbs.dtu.dk/services/BepiPred/
B cell prediction	iBCE-EL	http://thegleelab.org/iBCE-EL/
B cell prediction	ElliPro	http://tools.iedb.org/ellipro
CTL prediction	IEDB	http://tools.iedb.org/mhci/
Immunogenicity	IEDB	http://tools.immuneepitope.org/immunogenicity/
HTL prediction	IEDB	http://tools.iedb.org/mhcii/
TAP and proteasome	NetCTL 1.2	https://services.healthtech.dtu.dk/services/NetCTL-1.2/
IFN- γ prediction	IFNEpitope	https://webs.iiitd.edu.in/raghava/ifnepitope/application.php
Population coverage	IEDB	http://tools.iedb.org/population
Antigenicity	VaxiJen 2.0	http://www.ddg-pharmfac.net/vaxijen/VaxiJen/VaxiJen.html
Antigenicity	AntigenPro	https://selectpro.proteomics.ics.uci.edu/
Allergenicity	AllergenFP 1.0	http://ddg-pharmfac.net/AllergenFP/
Allergenicity	AllerTOP 2.0	https://www.ddg-pharmfac.net/AllerTOP/
Solubility	SolPro	https://selectpro.proteomics.ics.uci.edu/
Solubility	Protein-sol	https://protein-sol.manchester.ac.uk
Toxicity	ToxinPred	https://webs.iiitd.edu.in/raghava/toxinpred/index.html
Physicochemical properties	ExPASy ProtParam	https://web.expasy.org/protparam/
MHC cluster analysis	MHCcluster v2.0	https://services.healthtech.dtu.dk/services/MHCcluster-2.0/
Secondary structure	PSIPRED	http://bioinf.cs.ucl.ac.uk/psipred/
Secondary structure	SPOMA	https://prabi.ibcp.fr/htm/site/web/app.php/home
Secondary structure	Phyre2	http://www.sbg.bio.ic.ac.uk/~phyre2/html/page.cgi?id=index
3D structure	RoseTTAFold	https://robetta.bakerlab.org/
Structure refinement	GalaxyWEB	http://galaxy.seoklab.org/
Ramachandran plot	PROCHECK	https://saves.mbi.ucla.edu/
Z-score	ProSA-web	https://prosa.services.came.sbg.ac.at/prosa.php
Disulfide engineering	Design 2 v12.2	http://cptweb.cpt.wayne.edu/DbD2/
Protein prediction	Chimera V 1.13.1	https://www.cgl.ucsf.edu/chimera/olddownload.html
Protein docking	HADDOCK	https://wenmr.science.uu.nl
Protein docking	ClusPro 2.0	https://cluspro.bu.edu/login.php
Protein-protein interaction	PDBsum	https://www.ebi.ac.uk/thornton-srv/databases/pdbsum/
Protein interaction	LigPlot+	https://www.ebi.ac.uk/thornton-srv/software/LigPlus/
Molecular dynamic simulation	iMOD	http://imods.chaconlab.org
Codon optimization	JCat	http://www.jcat.de/
Immune simulation	C-ImmSim	https://kraken.iac.rm.cnr.it/C-IMMSIM/
Cloning	SnapGene	https://www.snapgene.com/
Search cavity	CB-Dock2	http://183.56.231.194:8001/cb-dock2/index.php

Immunogenic linear and conformational B cell epitope prediction.

**Figure 2:**
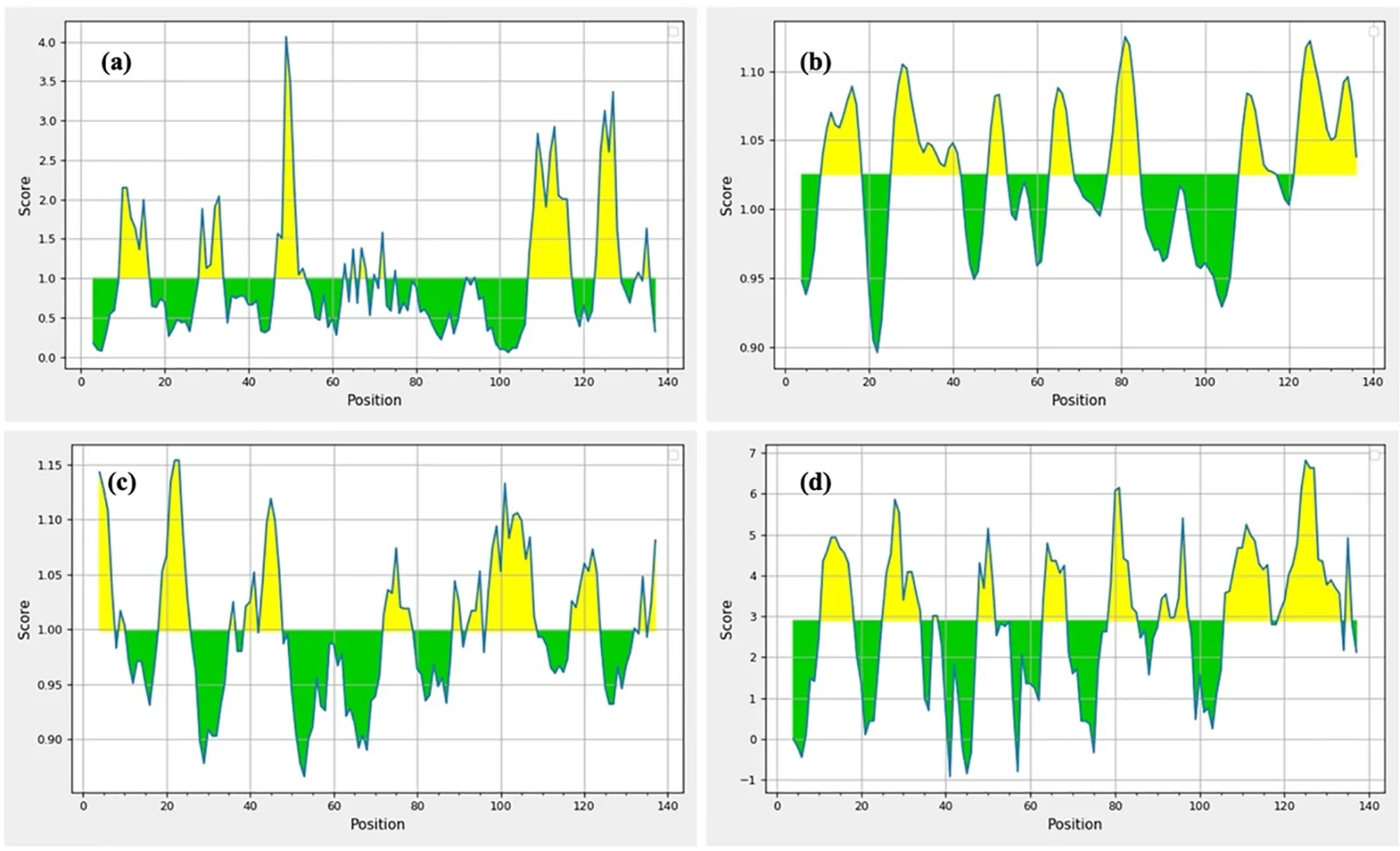
Validation of candidate B cell epitopes across multiple IEDB analytical servers, with favorable structural regions highlighted in yellow. **(A)** Surface accessibility profiling using the Emini prediction method. **(B)** Conformational flexibility assessment conducted via the Karplus & Schulz algorithm. **(C)** Evaluation of relative antigenic potential executed using the Kolaskar & Tongaonkar scale. **(D)** Hydrophilicity distribution mapped according to the Parker parameters.

**Figure 3:**
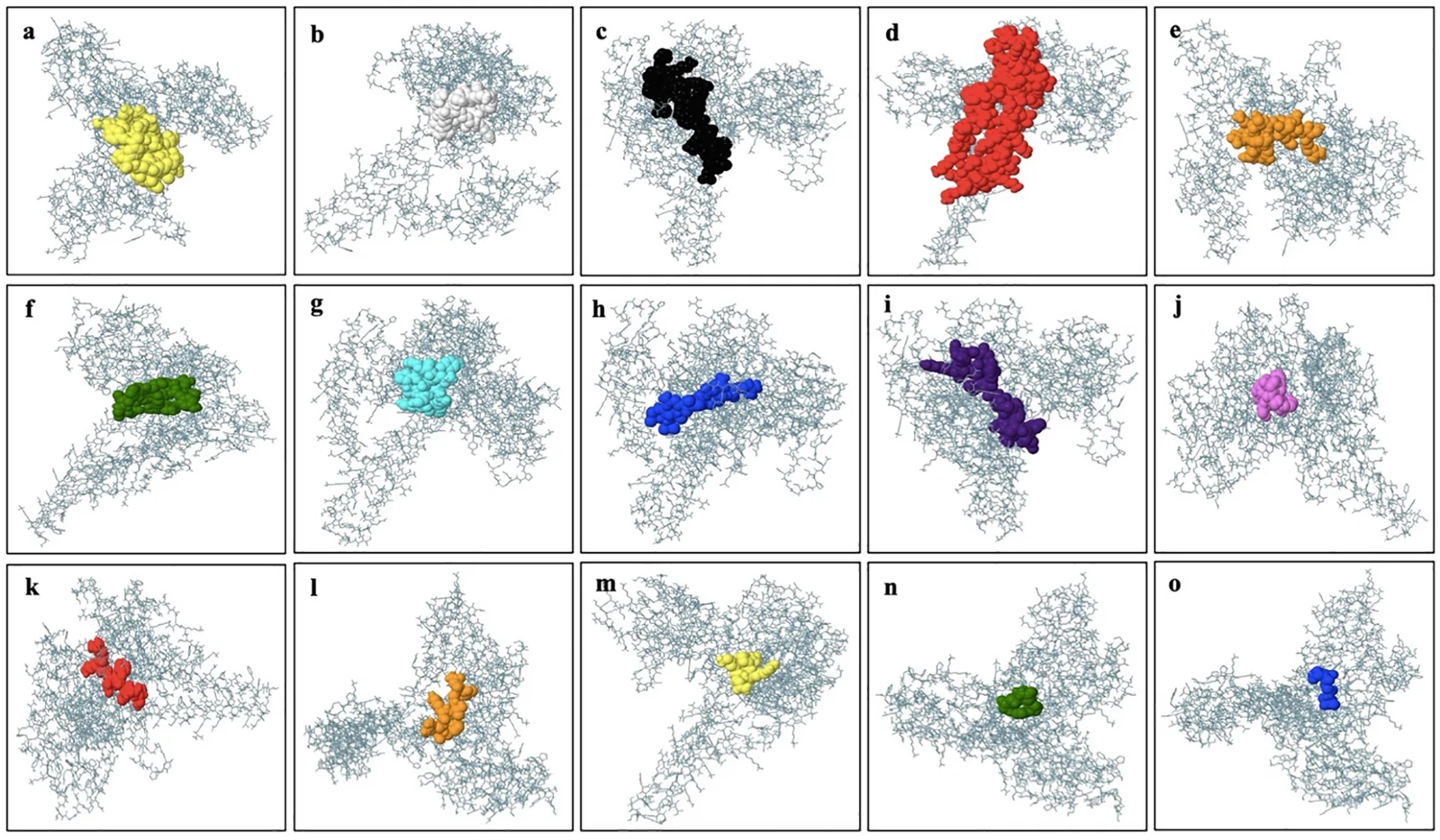
Structural mapping of predicted conformational (discontinuous) B cell epitopes within the MEV architecture. Epitope identification was executed utilizing the ElliPro platform. Panels **(A–O)** illustrate these configurations within the highly antigenic S polyprotein modeled in 3D. The main polyprotein backbone is represented by sky-blue stick structures, while the individual epitope sites are color-coded on the molecular surface.

Robust viral elimination depends on the simultaneous activation of both humoral and cellular arms of the adaptive immune system. B cell epitopes against Omicron S protein were identified using iBCE-EL, BepiPred 2.0, and IEDB. The epitopes detected by iBCE-EL that were missed by the other two servers were removed. To ensure their efficacy and encourage interaction with B cells, B cell peptides are positioned in solvent-favored regions of the antigen. Therefore, it was crucial to predict the surface accessibility of the protein sequence. Using the Emini surface accessibility scale, we profiled the S protein sequence to exclude interior, non-exposed epitopes (**Figure 2**). From this filtered dataset, we isolated twelve linear B cell peptides displaying high immunogenic potential (**Table 2**). The intrinsic flexibility, baseline antigenicity, and net hydrophilicity of these finalized epitopes were quantified via the Karplus & Schulz, Kolaskar & Tongaonkar, and Parker prediction metrics, respectively (**Figure 2**). Discontinuous (conformational) B-cell epitopes were predicted using the ElliPro server based on the 3D structure of the designed MEV candidate. ElliPro identified 15 conformational B-cell epitopes comprising a total of 498 amino acid residues, with protrusion index (PI) scores ranging from 0.500 to 0.773. The predicted conformational epitopes are illustrated on 3D structure of the MEV candidate in **Figure 3** and **Table 3**.

**Table 2:** Predicted linear B cell epitopes located on S glycoprotein.

Position	Peptide	Antigenicity	Allergenicity	Toxicity	GRAVY
13-28	*SQCVNLITRTQSYTNS*	0.4529	No	No	-0.581
30-35	*TRGVYY*	1.0433	No	No	-0.667
67-82	*VSGTNGTKRFDNPALP*	0.6131	No	No	-0.781
134-152	*NDPFLDVYQKNNKSWMESE*	0.4289	No	No	-1.5
172-185	*LMDLEGKEGNFKNL*	1.1915	No	No	-0.714
202-218	KHTPINLERDLPQGFSA	0.6742	No	No	-0.824
204-215	TPINLERDLPQG	0.5819	No	No	-0.892
242-258	*RSYLTPGDSSSGWTAGA*	0.4131	No	No	-0.6
650-663	*EYVNNSYECDIPIG*	0.8608	No	No	-0.514
667-687	*CASYQTQTKSHRRARSVASQS*	0.6669	No	No	-1.224
770-775	*QDKNTQ*	0.4997	No	No	-3.1
1030-1039	*LGQSKRVDFC*	1.8685	No	No	-0.33

**Table 3:** Predicted conformational B-cell epitopes of the designed MEV candidate identified using the ElliPro server.

Position	Peptide	Residues	Score
358-399	TQLKRRAAEIRASANLAATKGSGGSWFTALTQHGKEDLKIGY	42	0.843
189-204	WNRKRRKSKLKPFERL	16	0.825
308-331	PPTEPKGGSGGYFKIYSKHTPINL	24	0.781
437-498	FGRRGWLTYTGAIKLDDKDPNFKDQVILLNKQKKQQTVTLLPAADLGSGGHPNYDHDRMHTQ	62	0.749
146-164	RSVASQSGSGQDKNTQLGQ	19	0.727
119-135	NSYECDIPIGGSGCASY	17	0.682
283-295	YYLNSTPGSSKRG	13	0.673
228-237	RFNGINASVV	10	0.643
253-272	SGGNQRNALRITFSSPDDQI	20	0.635
100-108	PGDSSSGWT	9	0.605
166-174	KRVDFCGSG	9	0.581
40-46	TNGTKRF	7	0.547
422-426	KPRQK	5	0.543
53-57	GSGND	5	0.532
23-37	SGSGT	5	0.528

### CTL epitope prediction selection and immunogenicity assessment

To predict MHC-I-binding epitopes in the S and N proteins, the IEDB prediction method was evaluated. Leveraging the robust antigenicity of the target proteins, we identified 21 S-protein and 18 N-protein CTL epitopes. Each of these 39 identified peptides met the structural criteria of an 8–12 amino acid length necessary for efficient MHC Class I restriction (**Tables 4**, **5**). Further analysis was conducted on all of the nominated epitopes that were anticipated to be effective T cell binders.The epitope candidates were further assessed according to mean MHC-I processing values, immunogenicity, and global population coverage in different regions. The epitopes with the significant values were then examined across several SARS-CoV-2 variations as well as potential allergenicity, toxicity, and chemotoxicity. Furthermore, MHC-I epitopes were finalized because of their higher binding affinity. There were no overlapping MHC-I epitopes in the final screening process.

**Table 4:** CTL epitopes mapped within S glycoprotein utilizing the IEDB platform.

Position	Peptide	IC_50_	Antigenicity	Allergenicity	Toxicity	GRAVY	PCC	TAP
66-74	HVSGTNGTK	47.63	1.0956	No	No	-1.044	0.9438	0.506
72-80	GTKRFDNPA	23.11	0.4047	No	No	-1.5	0.6893	-0.74
86-94	*GVYFASTEK*	15.17	0.7112	No	No	-0.2	0.9698	0.609
197-206	FKIYSKHTPI	9.13	1.016	No	No	-0.36	0.9172	0.852
198-206	KIYSKHTPI	9.39	0.7455	No	No	-0.711	0.9172	0.955
260-269	AYYVGYLQPR	22.08	1.3309	No	No	-0.41	0.844	1.961
261-269	YYVGYLQPR	21.9	1.4692	No	No	-0.656	0.844	1.805
344-353	*ASVYAWNRKR*	24.77	0.6453	No	No	-1.16	0.773	2.017
453-462	*RKSKLKPFER*	23.63	0.6276	No	No	-2	0.8536	1.762
454-462	KSKLKPFER	7.66	1.0374	No	No	-1.722	0.8536	1.628
488-497	*LQSYGFRPTY*	4.99	0.7813	No	No	-0.75	0.9759	3.213
608-616	YQGVNCTEV	22.88	1.3957	No	No	-0.222	0.8962	0.205
622-631	*ADQLTPTWRV*	35.96	0.5883	No	No	-0.56	0.2681	1.287
672-681	*TQTKSHRRAR*	32	0.8608	No	No	-2.45	0.2032	1.484
673-681	QTKSHRRAR	9.39	0.9058	No	No	-2.644	0.2032	1.472
675-683	KSHRRARSV	11.35	1.0771	No	No	-1.8	0.6683	0.49
675-684	KSHRRARSVA	20.34	1.0846	No	No	-1.44	0.7689	0.49
752-761	*YGSFCTQLKR*	21.05	1.213	No	No	-0.6	0.8366	1.406
897-905	*QMAYRFNGI*	10.02	0.6803	No	No	-0.244	0.5063	0.684
1169-1177	*NASVVNIQK*	14.46	0.8372	No	No	-0.056	0.9075	0.548
1203-1212	*EQYIKWPWYI*	21.01	1.1122	No	No	-0.79	0.7142	2.981

To map candidate CTL epitopes from the S glycoprotein, we executed predictions via the IEDB platform. Selection criteria required candidates to demonstrate strong antigenicity profiles, optimal IC_50_ values, and low dissociation constants (nM) for MHC-I HLA alleles. Furthermore, we assessed processing efficiency by simulating TAP transport and proteasomal C-terminal cleavage. Every candidate was also cross-referenced for sequence conservation, and the final vaccine components are highlighted using italicized text.

**Table 5:** CTL epitopes mapped within N phosphoprotein utilizing the IEDB platform.

Position	Peptide	IC_50_	Antigenicity	Allergenicity	Toxicity	GRAVY	PCC	TAP
8-17	*NQRNALRITF*	37.01	1.1293	No	No	-0.73	0.9075	2.801
75-84	*SSPDDQIGYY*	33.46	0.4533	No	No	-1.22	0.9744	3.001
97-105	KMKDLSPRW	83.99	1.7462	No	No	-1.489	0.9739	1.109
97-106	KMKDLSPRWY	62.64	1.5032	No	No	-1.47	0.7173	2.946
99-108	KDLSPRWYFY	51.77	0.9981	No	No	-1.12	0.9742	2.725
101-109	LSPRWYFYY	48.64	1.2832	No	No	-0.567	0.9698	2.952
101-110	*LSPRWYFYYL*	37.51	1.3486	No	No	-0.13	0.9662	2.952
102-110	SPRWYFYYL	6.32	0.734	No	No	-0.567	0.9781	0.75
102-111	SPRWYFYYLG	77.58	0.9336	No	No	-0.55	0.9777	0.75
190-198	SSRNSTPGS	32.96	1.2424	No	No	-1.544	0.1416	-2.105
193-201	*NSTPGSSKR*	37.12	0.8958	No	No	-1.889	0.9409	1.524
259-267	RTATKAYNV	50.96	0.6395	No	No	-0.756	0.6527	0.552
261-269	ATKAYNVTQ	99.22	0.6635	No	No	-0.644	0.4374	0.013
318-327	*GMEVTPSGTW*	98.83	0.6967	No	No	-0.29	0.9714	0.844
357-366	YKTFPPTEPK	11.53	0.7633	No	No	-1.6	0.9629	0.61
358-366	*KTFPPTEPK*	6.28	0.7571	No	No	-1.633	0.9629	0.66
358-367	KTFPPTEPKK	11.43	0.7657	No	No	-1.86	0.9394	0.697
382-391	*RQKKQQTVTL*	63.96	0.7542	No	No	-1.62	0.9723	1.147

Computational identification of CTL epitopes within the N phosphoprotein was performed via IEDB analysis. Selection criteria dictated that candidate sequences demonstrate strong antigenicity, low IC_50_ values, and high-affinity binding (nM) to MHC-I HLAs. Processing criteria, specifically TAP transport and proteasomal C-terminal cleavage capacities, were calculated alongside strain conservation metrics. Italicized font indicates the final selections chosen for MEV.

The immunogenicity scores for the selected MHC-I epitopes and the modification of T cell epitopes were predicted using the European Molecular Biology Open Software Suite (EMBOSS 6.4.0) and the IEDB server. High-scoring epitopes were extracted via the IEDB immunogenicity tool. The selection framework combined antigenicity and immunogenicity values into a cohesive fitness metric, successfully highlighting the leads most likely to stimulate a potent immune reaction.

### HTL epitope prediction selection

HTL are indispensable for adaptive immunity, driving B cell antibody production and facilitating CTL mediated target cell lysis. Once activated by antigen-bearing APCs, naive HTL differentiate into the distinct functional phenotypes required to coordinate a targeted, pathogen-specific immune response [26]. Given the significance of HTL epitopes in this context, we also anticipated HTL epitopes that would support B and T cell epitopes in eliciting strong immunological feedback. IEDB was used to map the HTL epitopes on the S and N proteins. Analyzed using CTL-like criteria, 7 S protein and 22 N protein immunogenic HTL epitopes were discovered (**Tables 6**, **7**). The selected 14- to 16-mer epitopes demonstrated highly promiscuous binding profiles across multiple HLA-II alleles. These HTL candidates were then curated using population coverage metrics to ensure maximum genetic compatibility across diverse geographic regionsThe top-scoring epitopes were subsequently assessed for characteristics such as allergenicity, toxicity, chemotoxicity, their capacity to stimulate cytokine activation, and their conservation across many SARS-CoV-2 variants. MHC-II epitopes that overlapped were treated as distinct entities. To complete the screening process, each MHC-II epitope was evaluated for its capacity to stimulate IFN-γ production.

**Table 6:** HTL epitopes mapped within S glycoprotein utilizing the IEDB platform.

Position	Peptide	IC_50_	Antigenicity	Allergenicity	Toxicity	GRAVY	IFNepitope SVM^b^
195-209	*GYFKIYSKHTPINLE*	8.5	1.1005	No	No	-0.567	Positive
233-247	RFQTLLALHRSYLTP	7.1	0.547	No	No	-0.067	Positive
262-276	YVGYLQPRTFLLKYN	4.1	0.5078	No	No	-0.24	Positive
263-277	*VGYLQPRTFLLKYNE*	5.7	0.553	No	No	-0.387	Positive
747-761	*NLLLQYGSFCTQLKR*	10.9	1.0102	No	No	-0.107	Positive
757-771	TQLKRALTGIAVEQD	13.7	0.4809	No	No	-0.287	Positive
1010-1024	*RAAEIRASANLAATK*	12.7	0.5709	No	No	-0.153	Positive

To discover viable HTL epitopes, we leveraged the predictive algorithms of the IEDB platform. We screened the resulting surface glycoprotein sequences by analyzing their antigenicity rates, percentile ranks, and MHC-II HLA binding values (nM). Functional validation involved predicting IFN-γ induction potential via an SVM-based approach. Furthermore, we integrated a combined motif and SVM framework to contrast IFN-γ secretion against alternative cytokines, while filtering for sequence conservation. The specific candidates selected for the final MEV candidate are highlighted using italicized font.

**Table 7:** HTL epitopes mapped within N phosphoprotein utilizing the IEDB platform.

Position	Peptide	IC_50_	Antigenicity	Allergenicity	Toxicity	GRAVY	IFNepitope SVM^b^
46-60	TASWFTALTQHGKED	13.4	0.4491	No	No	-0.773	Positive
47-61	ASWFTALTQHGKEDL	13.9	0.4116	No	No	-0.473	Positive
48-62	*SWFTALTQHGKEDLK*	21.9	0.6641	No	No	-0.853	Positive
80-94	QIGYYRRATRRIRGG	3.5	0.4614	No	No	-1.313	Positive
81-95	*IGYYRRATRRIRGGD*	3.5	0.6649	No	No	-1.313	Positive
102-116	SPRWYFYYLGTGPEA	34.1	0.8767	No	No	-0.66	Negative
103-117	PRWYFYYLGTGPEAG	15.4	0.8083	No	No	-0.633	Negative
104-118	RWYFYYLGTGPEAGL	9.7	0.7505	No	No	-0.273	Negative
105-119	WYFYYLGTGPEAGLP	11.9	0.7188	No	No	-0.08	Negative
244-258	*TKKSAAEASKKPRQK*	49.8	0.6677	No	No	-1.967	Positive
254-268	KPRQKRTATKAYNVT	44	0.4122	No	No	-1.66	Positive
261-275	*ATKAYNVTQAFGRRG*	10	0.7146	No	No	-0.733	Positive
262-276	TKAYNVTQAFGRRGP	10.9	0.5975	No	No	-0.96	Positive
263-277	KAYNVTQAFGRRGPE	12.9	0.6104	No	No	-1.147	Positive
324-338	SGTWLTYTGAIKLDD	5.9	0.6215	No	No	-0.193	Positive
325-339	GTWLTYTGAIKLDDK	6.6	0.9934	No	No	-0.4	Positive
326-340	TWLTYTGAIKLDDKD	7.2	1.2416	No	No	-0.607	Positive
327-341	*WLTYTGAIKLDDKDP*	8.7	1.2787	No	No	-0.667	Positive
341-355	*PNFKDQVILLNKHID*	40.3	0.988	No	No	-0.433	Positive
342-356	NFKDQVILLNKHIDA	44.7	0.7693	No	No	-0.207	Positive
353-367	HIDAYKTFPPTEPKK	37.5	0.4004	No	No	-1.353	Positive
383-397	*QKKQQTVTLLPAADL*	23.2	0.7662	No	No	-0.373	Positive

Informatics tracking of HTL epitopes for the N phosphoprotein relied on the IEDB server, with selection determined by MHC-II HLA binding strengths (nM), percentile ranks, and antigenicity rates. We validated the functional capacity of these targets through an SVM methodology focused on IFN-γ induction. Furthermore, a dual motif/SVM strategy contrasted their specific IFN-γ potential with other cytokine pathways. After confirming sequence conservation, the highly immunogenic epitopes integrated into the final formulation were marked using italicized text.

### Highly immunogenic epitope selection

B and T cell epitopes were characterized biophysically to design an MEV capable of eliciting a balanced adaptive response. While CTLs drive antigen-specific cytotoxicity, HTLs mobilize B cells and macrophages. Antigen-stimulated B cells subsequently mature into plasma cells, generating the antibody repository responsible for humoral defense. To overcome the natural waning of circulating antibodies over extended periods, the engineered vaccine intentionally prioritizes potent cell-mediated memory pathways alongside the humoral axis. On the other hand, the cell-mediated immune response provides stronger and more durable protection by generating antiviral cytokines and, in particular, identifying and destroying infected cells (51). In this project, S and N proteins were used as models for identifying epitopes. High-potency T cell epitopes and B cell sequences longer than seven residues were advanced to a stringent multi-step screening pipeline. Epitope verification was based on maximizing antigenicity and strain conservation while explicitly screening out allergenic, toxic, or human-homologous peptides. The final MEV candidate was constructed using the epitopes that met these criteria (**Table 8**). After their potential was evaluated, only HTL epitopes eligible of inducing cytokine production were added to the vaccine development process.

**Table 8:** Consolidated list of finalized S and N protein epitopes (B cell, CTL, and HTL) utilized in the vaccine formulation.

Cell type	Peptide	Antigenicity	Allergenicity	Toxicity	GRAVY
B Cell					
13-28	SQCVNLITRTQSYTNS	0.4529	No	No	0.581
30-35	TRGVYY	1.0433	No	No	-0.667
67-82	VSGTNGTKRFDNPALP	0.6131	No	No	-0.781
134-152	NDPFLDVYQKNNKSWMESE	0.4289	No	No	-1.5
172-185	LMDLEGKEGNFKNL	1.1915	No	No	-0.714
242-258	RSYLTPGDSSSGWTAGA	0.4131	No	No	-0.6
650-663	EYVNNSYECDIPIG	0.8608	No	No	0.514
667-687	CASYQTQTKSHRRARSVASQS	0.6669	No	No	-1.224
770-775	QDKNTQ	0.4997	No	No	-3.1
1030-1039	LGQSKRVDFC		No	No	-0.33
CTL					
87-94	VYFASTEK	0.7112	No	No	-0.2
344-353	ASVYAWNRKR	0.6453	No	No	-1.16
453-462	RKSKLKPFER	0.6276	No	No	-2
488-497	LQSYGFRPTY	0.7813	No	No	-0.75
622-631	ADQLTPTWRV	0.5883	No	No	-0.56
897-905	QMAYRFNGI	0.6803	No	No	-0.244
1169-1177	NASVVNIQK	0.8372	No	No	-0.056
1203-1212	EQYIKWPWYI	1.1122	No	No	-0.79
8-17	*NQRNALRITF*	1.1293	No	No	-0.73
75-84	*SSPDDQIGYY*	0.4533	No	No	-1.22
101-110	*LSPRWYFYYL*	1.3486	No	No	-0.13
193-201	*NSTPGSSKR*	0.8958	No	No	-1.889
318-327	*GMEVTPSGTW*	0.6967	No	No	-0.29
358-366	*KTFPPTEPK*	0.7571	No	No	-1.633
HTL					
195-209	GYFKIYSKHTPINLE	1.1005	No	No	-0.567
263-277	VGYLQPRTFLLKYNE	0.553	No	No	-0.387
747-761	NLLLQYGSFCTQLKR	1.0102	No	No	-0.107
1010-1024	RAAEIRASANLAATK	0.5709	No	No	-0.153
48-62	*SWFTALTQHGKEDLK*	0.6641	No	No	-0.853
81-95	*IGYYRRATRRIRGGD*	0.6649	No	No	-1.313
244-258	*TKKSAAEASKKPRQK*	0.6677	No	No	-1.967
261-275	*ATKAYNVTQAFGRRG*	0.7146	No	No	-0.733
327-341	*WLTYTGAIKLDDKDP*	1.2787	No	No	-0.667
341-355	*PNFKDQVILLNKHID*	0.988	No	No	-0.433
383-397	*QKKQQTVTLLPAADL*	0.7662	No	No	-0.373

The finalized MEV candidate comprises a selective blend of B cell, CTL, and HTL epitopes mapped through various computational servers. Sequences were screened using stringent computational criteria, including high predicted antigenicity, predicted non-toxicity, predicted non-allergenicity, and negative GRAVY values, to identify promising vaccine candidates for further experimental evaluation. Only candidates exhibiting superior immunogenic potential were retained. Epitopes belonging to the N protein are explicitly indicated in italics.

### Proteasomal cleavage/TAP transport

We deployed the NetCTL1.2 algorithm to screen the S and N structural antigens, focusing specifically on MHC Class I processing pathways. This approach accounts for both proteasomal cleavage kinetics and TAP-mediated transport two pivotal phases in classical antigen presentation (28). The individual TAP transport profiles and proteasomal processing scores are detailed explicitly in **Tables 3**, **4**. From this refined pool, we extracted the top-tier CTL epitopes based on prioritized immunogenicity rankings to assemble the core of the MEV.

### IFN-γ inducing epitope

IFN-γ drives effective antiviral immunity by stimulating innate effectors like NK cells and macrophages, while also enhancing MHC-mediated antigen presentation. Accordingly, candidate HTL epitopes were selected based on their verified ability to trigger IFN-γ production and their compatibility with MHC-II restriction profiles necessary for helper T cell priming. Several epitopes were removed during the screening stage because they failed to produce IFN-γ production (29). As detailed in **Tables 6**, **7**, a total of 29 HTL epitopes demonstrated a dual positive profile for both baseline immunogenicity and IFN-γ induction. Conversely, candidate sequences predicted to be non-inducers of IFN-γ were systematically excluded from further downstream analysis.

### Globally population coverage

**Figure 4:**
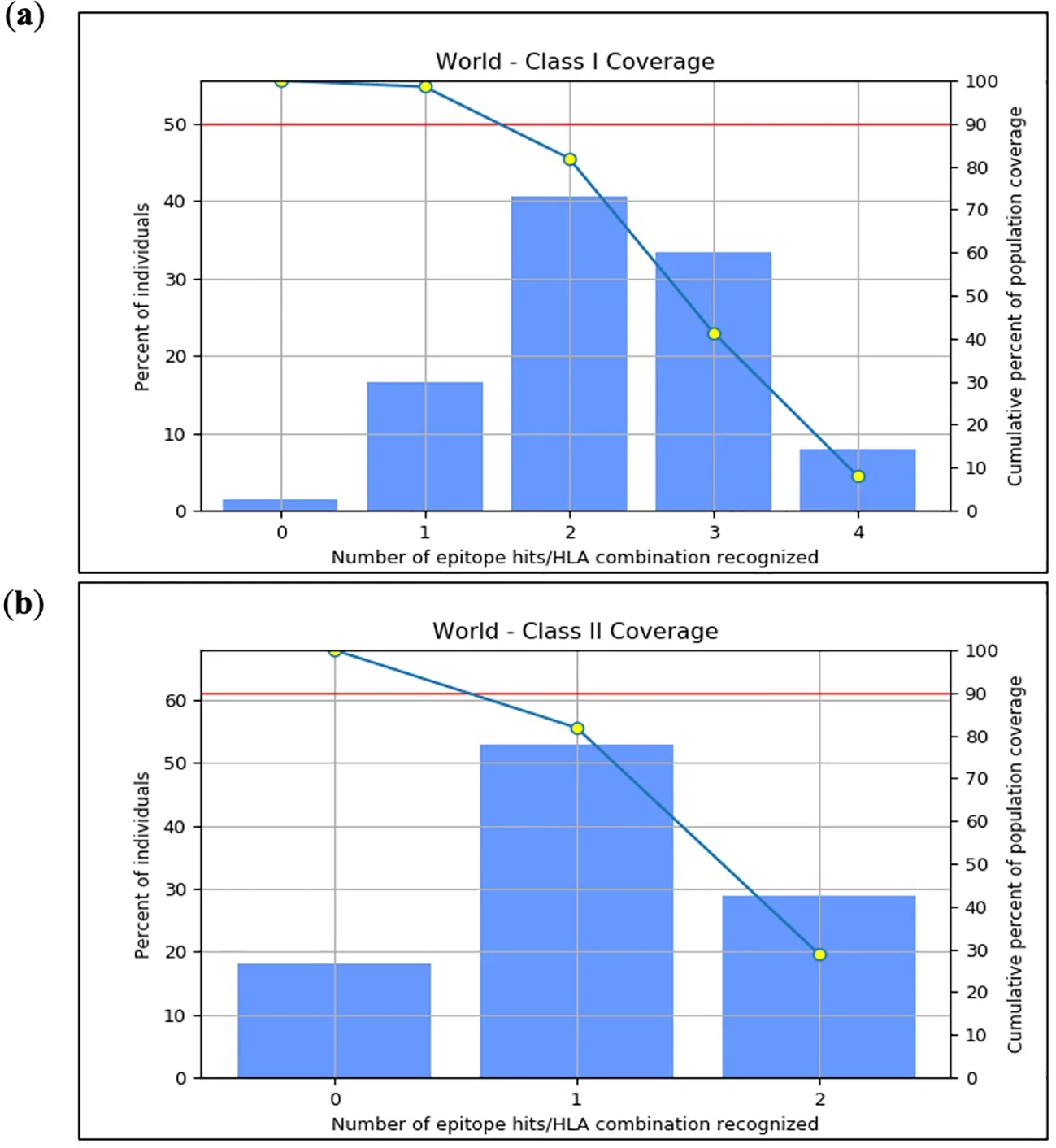
To evaluate the global distribution of our selected epitopes, we calculated the total population coverage percentages based on their respective HLA-binding alleles. **(A)** Consequently, the epitopes restricted to MHC-I alleles demonstrate an extensive worldwide population coverage of 98.55%. **(B)** In comparison, the MHC-II restricted epitope candidates yield a global population coverage of 81.81%.

The population coverage was assessed using Population Coverage tool based on the worldwide population coverage of specific CTL and HTL epitopes and their binding alleles. Globally, HTL epitopes (81.81%) and CTL epitopes (98.55%) of the S and N proteins gave a high percentage of population coverage. As illustrated in **Figure 4**, the MHC Class I and Class II epitopes derived from the S and S antigens exhibited the highest overall demographic distribution. When evaluated in tandem, these combined MHC classes yield a projected global population coverage of 97.38%, which is noteworthy for its potential on a worldwide scale. Consequently, the final epitope selection carries the potential to elicit protective immunity across the vast majority of individuals worldwide, regardless of geographic distribution.

### Antigenicity, allergenicity and solubility analysis

The antigenic potential of the selected B cell, MHC Class I, and MHC Class II epitopes was rigorously validated by cross-referencing predictions from both the VaxiJen v2.0 and AntigenPRO servers. For the final MEV candidate, epitopes with significant antigenic potential were selected. AllergenFP 1.0 and AllerTOP 2.0 were the servers utilized to forecast allergenicity; only non-allergenic epitopes were taken into consideration for the vaccine. Two servers, SolPro and Protein-sol, were used to predict each epitope’s solubility. To select the higher solubility profile, the final epitope design was carried out under the guidance of solubility criteria. The immunogenic parameter information for each epitope is shown in **Tables 2**-**8**.

### Toxicity and physicochemical characteristics analysis

The ability of epitopes to provoke T cell responses is determined by their immunogenicity. The shortlisted epitopes underwent immunogenicity prediction. The potential of epitopes to stimulate humoral and cellular responses increases with the immunogenicity score. Toxicity was predicted using the ToxinPred service. The ExPASy ProtParam server was also used to predict a number of physicochemical properties, including as hydropathicity, charge, half-life, instability index, pI, and molecular weight. Overlapping B cell, CTL, and HTL peptide sequences under 20 residues in length were systematically screened for toxic characteristics utilizing the ToxinPred platform. Non-toxic peptides were found and added to the final MEV candidate following a battery of assessment procedures. The adjuvants and the complete vaccine sequence were examined for the presence of additional harmful peptides. To verify their harmless status, the remaining subunits and the HisTag underwent additional analysis utilizing the ToxinPred SVM prediction mode. To ensure optimal aqueous solubility, we evaluated the hydropathicity of each peptide, prioritizing candidates with negative values that reflect true hydrophilic characteristics. The finalized profiles regarding the physicochemical attributes and toxicity parameters for individual epitopes are summarized across **Tables 2**-**8**.

### MHC restriction cluster evaluation of immunogenic epitopes

**Figure 5:**
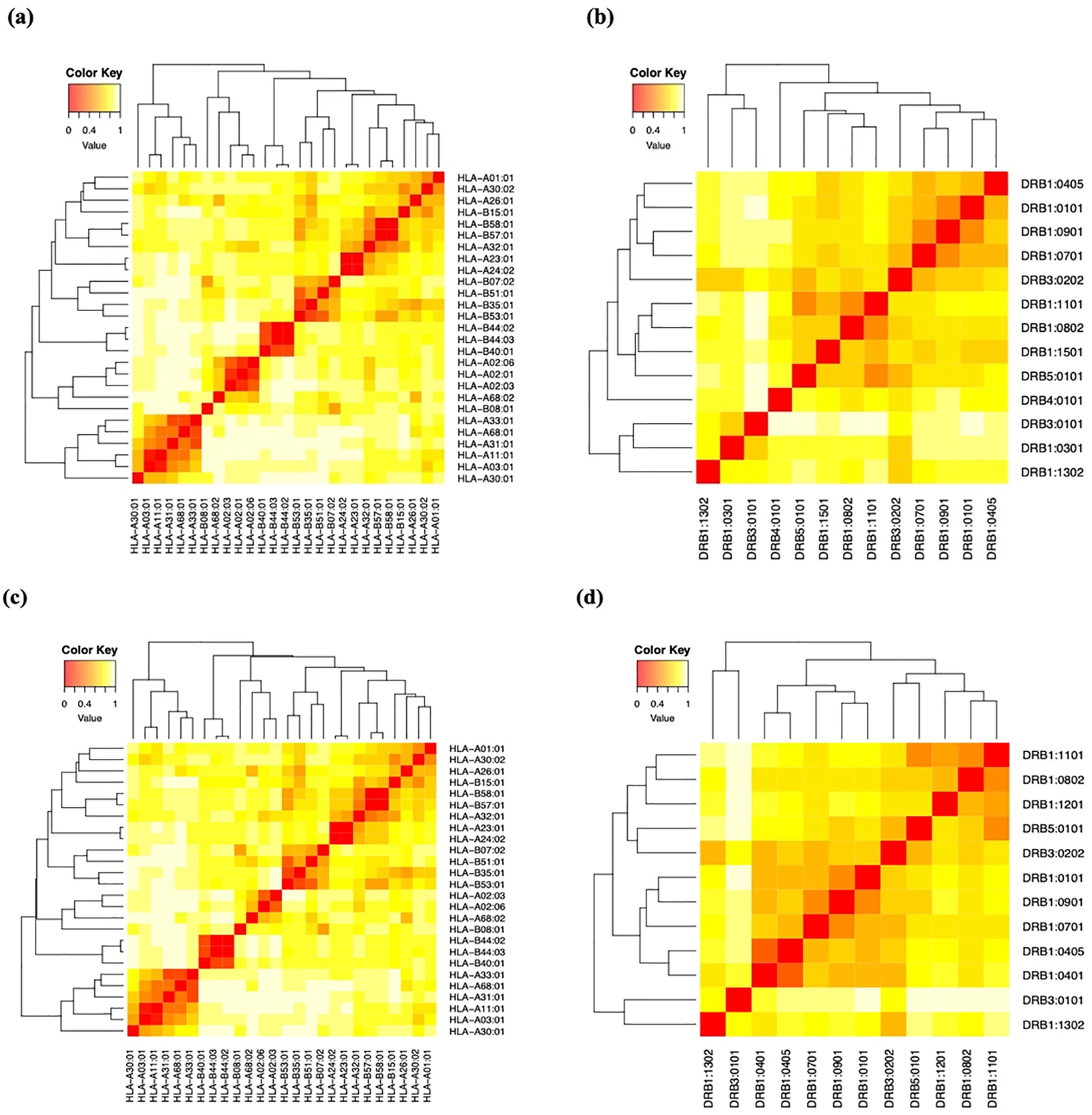
Dual-MHC class cluster analysis represented via hierarchical heatmaps. **(A, B)** Evaluation of MHC-I and MHC-II allele groupings utilizing the primary S epitope candidates from the final design. **(C, D)** Parallel cluster analysis conducted for MHC-I and MHC-II molecules using the high-potential N protein epitopes embedded within the MEV architecture.

The detected MHC-I/II epitopes were compared to MHC-restricted alleles using the MHCclusters program, and the epitopes found in T cells were validated by their appropriate peptide. The findings of the evaluation of the interacting alleles are used to annotate the interaction between MHC-I/II and HLAs as a heat map for S and N proteins (**Figures 5A–C**). The epitopes were categorized according to how well they associated with HLA; strong associations were displayed in red, and weak links were displayed in yellow.

### Modeling of MEV candidate

**Figure 6:**
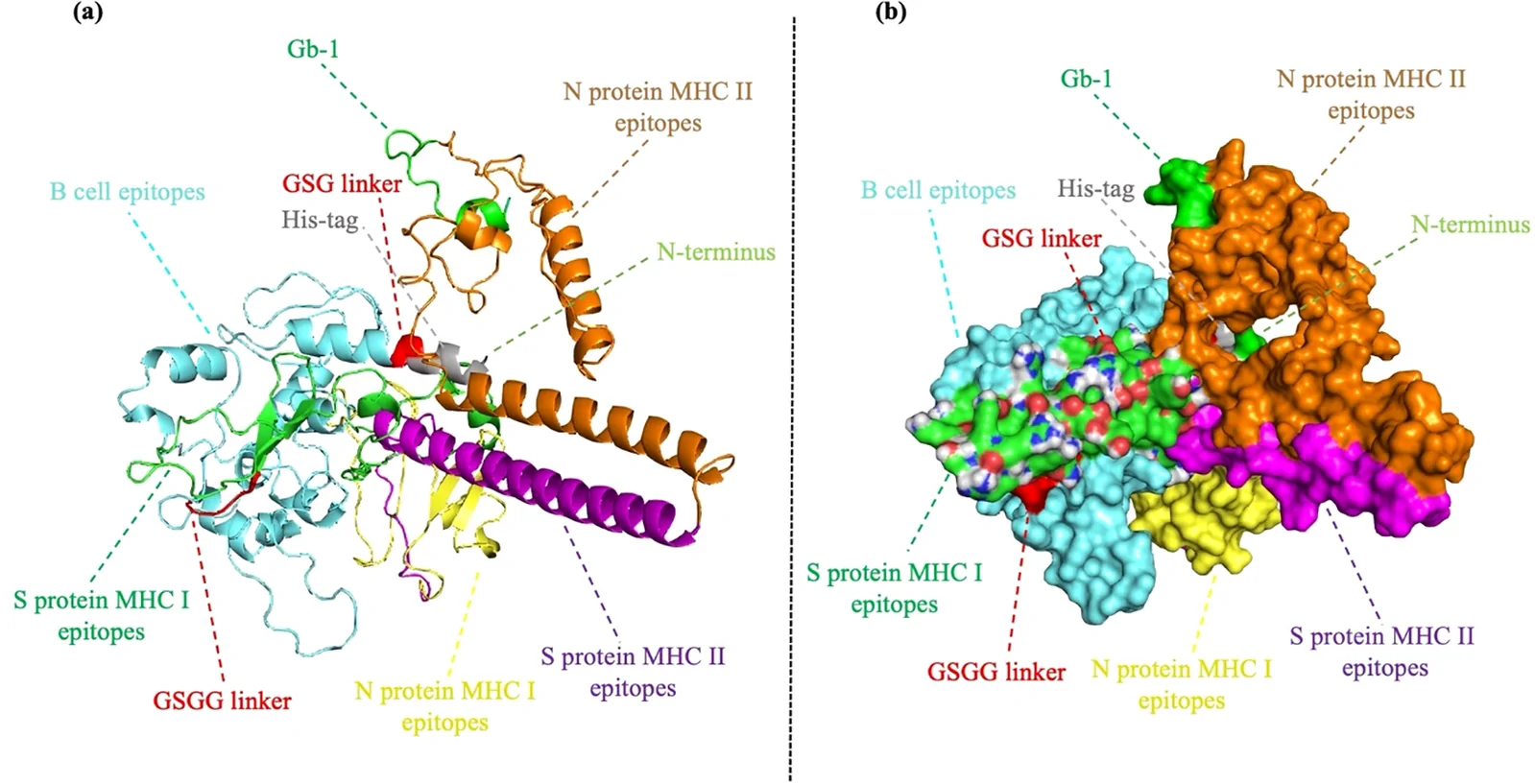
Modeling and localized mapping of immunodominant B cell and T cell epitopes within the finalized vaccine architecture. RoseTTAFold was utilized to determine the 3D structures and positions of the core S and N epitopes. **(A)** Linear sequences and topographic locales of the immunodominant segments are depicted in a cartoon representation. **(B)** The spatial geometries are rendered in 3D using PyMOL 2.3.4, highlighting the surface exposure patterns on the fully folded MEV candidate.

**Figure 7:**
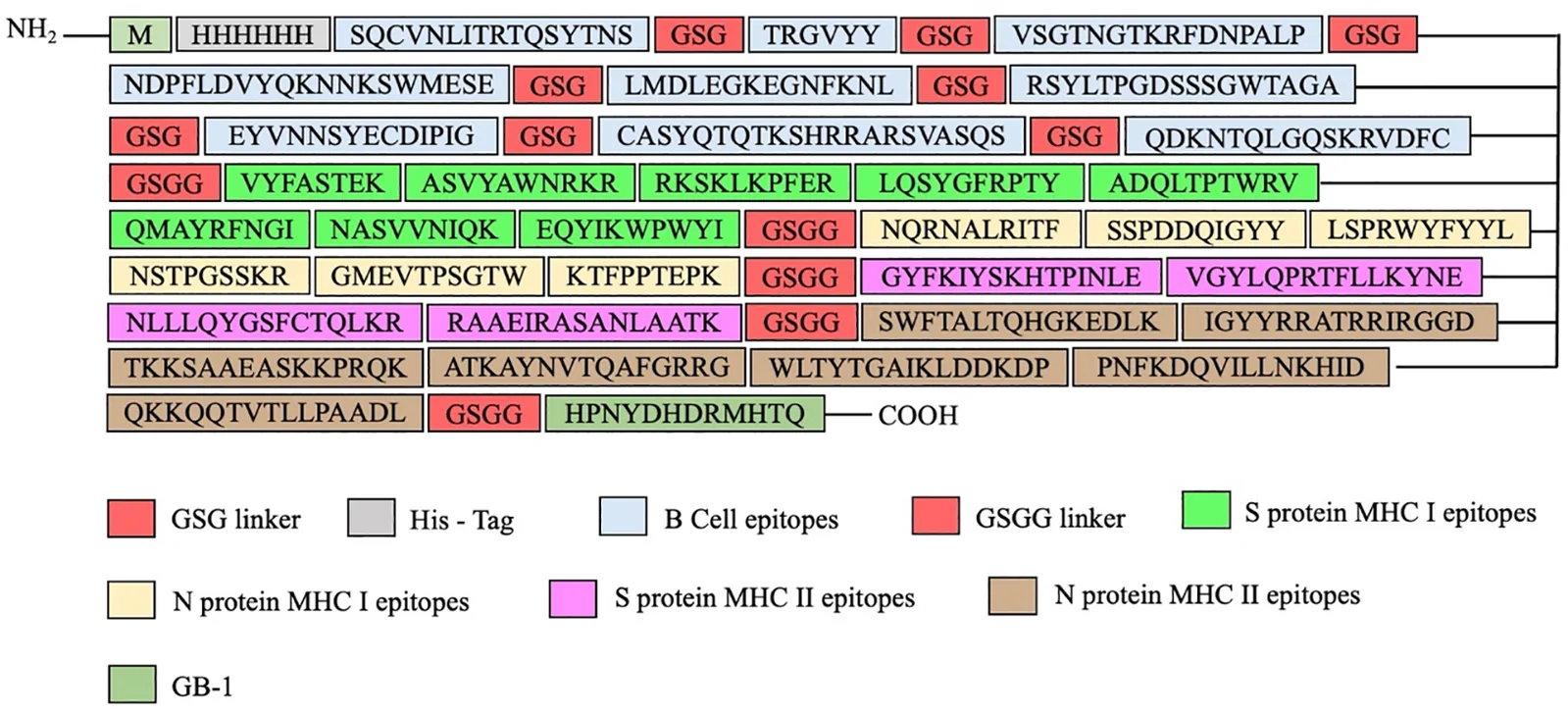
Conceptual diagram showing the linear alignment of epitopes, linkers, and adjuvants in the MEV candidate. The formulation joins a His-Tag to B cell epitopes through a short GSG sequence. The B cell epitopes are coupled to CTL epitopes via GSGG linker, followed by the insertion of GSGG linkers to bridge the CTL and HTL fragments. The construct terminates with the HTL epitopes attached to the Gb-1 using a final GSGG linker.

The criteria specified for isolating top-tier candidates state that MEV candidate formulation depended on a stringent vetting process. To be integrated, individual epitopes had to concurrently display elevated antigenicity, broad S and N strain conservation, and cytokine-inducing potential, while explicitly proving to be non-toxic, non-allergenic, and non-homologous to the human proteome. In order to effectively stimulate the innate immune system and elicit an immunological response, these epitopes showed the best promise for inclusion in the vaccine’s formulation. In this investigation, the SARS-CoV-2 proteins S and N were investigated in order to develop an epitope-based vaccine. Although antimicrobial peptides play documented roles in regulating innate and adaptive immunity, our design pipeline focused exclusively on antigen-specific viral epitopes. Following multi-parameter immunoinformatic evaluations, we selected 10 B cell, 11 HTL, and 14 CTL epitope leads as the foundational components for the MEV candidate. The resultant immunodominant epitopes are shown in a 3D model in **Figure 6**. The final immunogenic epitopes were coupled using specific linkers: the GSG linker was used to link the B cell epitopes, while the GSG and GSGG linkers were used to link the B cell and CTL epitopes. GSGG linkers were used to connect the C-terminal of GB-1 to the HTL and CTL epitopes. Following the proper combination and randomization, a vaccine design of 498 amino acid residues was created. The final MEV sequence is shown in **Figure 7**.

### Antigenicity, allergenicity and physicochemical parameters of MEV candidate

Based on antigenicity and allergenicity prediction tools, immunoinformatics analyses predicted that the SARS-CoV-2 proteins S and N MEV candidate has high antigenicity and is non-allergenic, indicating its potential to provoke strong immune feedback without causing adverse allergy reactions. The ProtParam server predicted MW and pI of the MEV to be 55.47 kDa and 9.84, respectively. The theoretical pI of 9.84 indicates that the MEV candidate is basic (alkaline) in nature, providing a physicochemical characteristic relevant to its computational evaluation. The aliphatic index (AI) of the MEV candidate was 53.51, and its chemical formula was C_2448_H_3761_N_721_O_739_S_11_. The residues’ charge distribution showed that 69 were positively charged and 36 were negatively charged. In the same way, the protein’s extinction coefficient in water was determined to be 99700 M^-^¹ cm^-^¹at 280 nm. A half-life of more than 10 hours in *E. coli* culture and 30 hours in human reticulocytes was measured by ProtParam, indicating stability across expression platforms. With an instability rating of 39.59, the suggested protein was determined to be stable. The proposed vaccine’s GRAVY value was found to be − 0.851, indicating that temperature variations might not cause instability in the suggested protein. In the same way, a negative GRAVY score suggests that the protein is hydrophilic and may result in better interactions with surrounding water molecules. These results show that the proposed vaccine formulation could be a helpful strategy for preventing SARS-CoV-2. The physicochemical properties of the designed vaccine are summarized in **Table 9**.

**Table 9:** Theoretical physicochemical properties of the engineered MEV candidate.

Features	Values
Sequence length	498 aa
Molecular weight	55.47 KDa
Formula	C_2448_H_3761_N_721_O_739_S_11_
Antigenicity	0.8437
Theoratical pI	9.84
Total negatively charged residues	36
Total positively charged residues	69
Total number of atoms	7680
Extinction of coffiecients	99700
Instability index	39.59
Aliphatic index	53.51
GRAVY	-0.851

### Evaluation of secondary structure of MEV candidate

**Figure 8:**
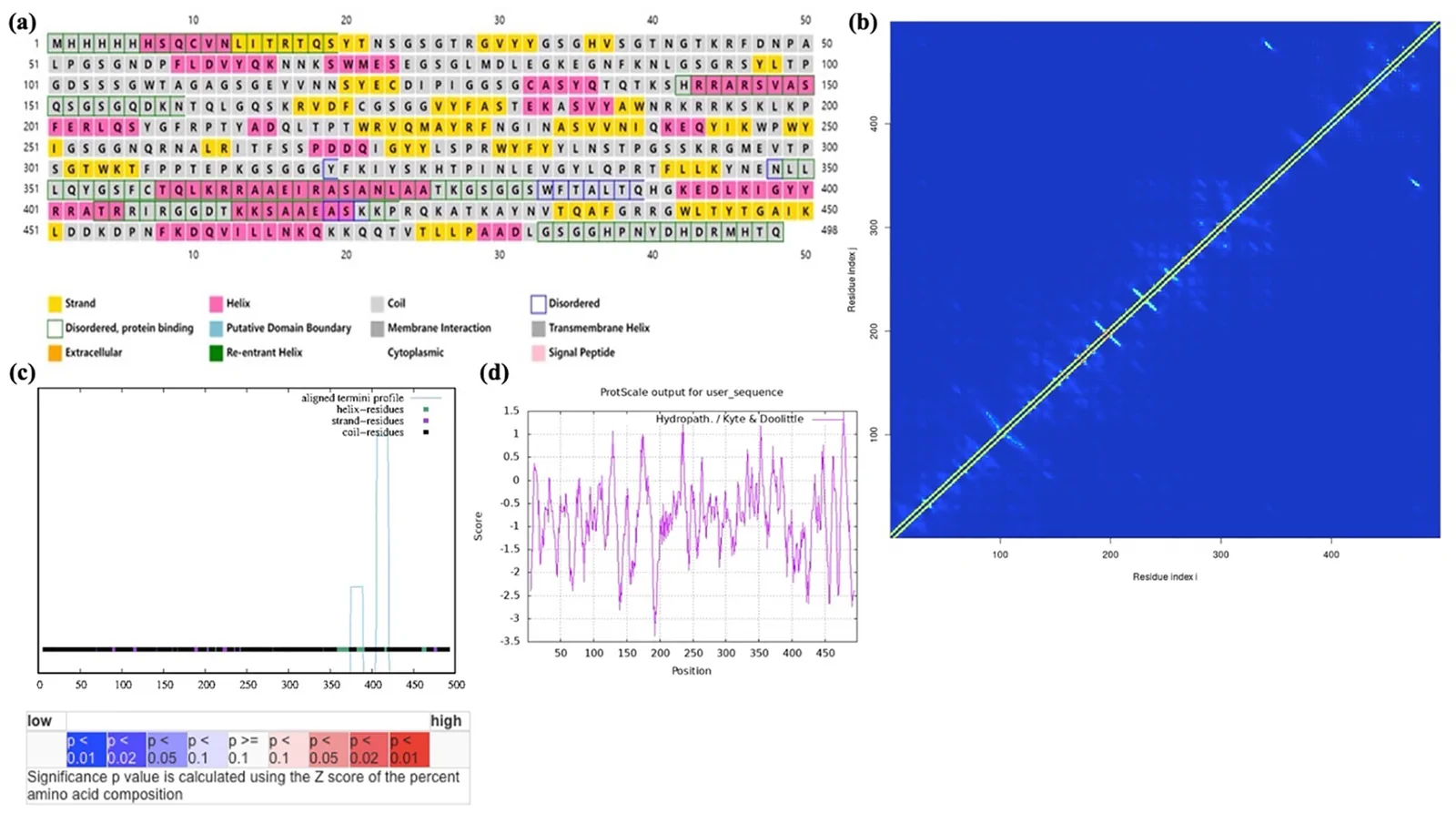
Structural modeling and hydropathy analysis of the engineered vaccine assembly. **(A)** Secondary structure mapping showing the exact positioning of helical, strand, and coiled domains across the amino acid sequence. **(B)** Continuous contact matrix plot visualizing localized residue index interactions and tertiary folding constraints. **(C)** Terminal alignment profiling and statistical significance thresholds evaluated across the peptide matrix. **(D)** Hydrophobicity index curve mapped according to the Kyte & Doolittle scale, reflecting the overall baseline solubility and hydropathic behavior of the sequence.

**Figure 9:**
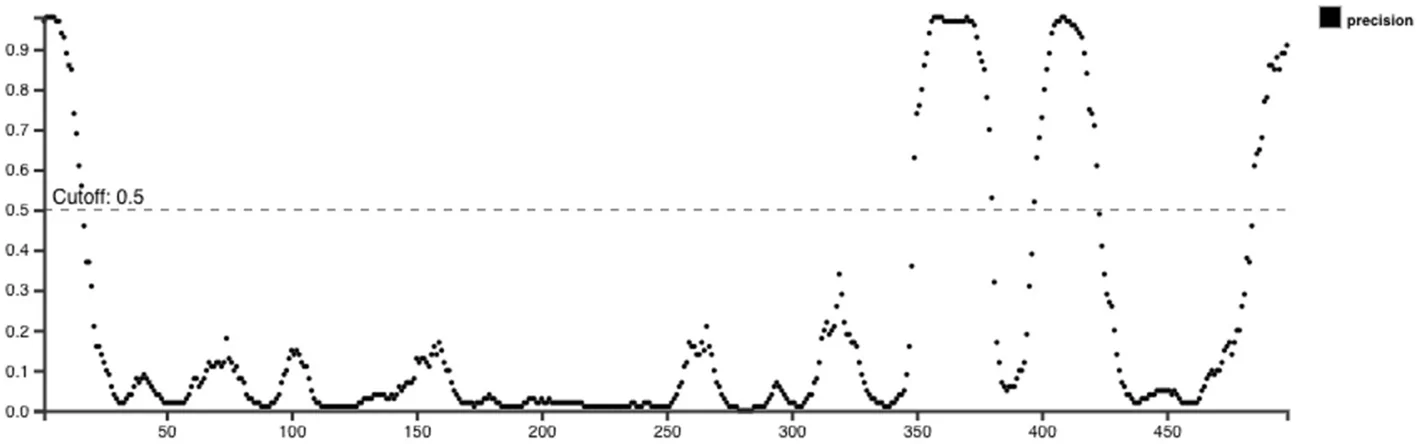
Intrinsic disorder within the secondary structure framework was mapped utilizing the PSIPRED platform. This analysis precisely identified disordered regions across the primary amino acid sequence. Based on the prediction parameters, residues are classified as disordered when the confidence curve exceeds the established threshold cutoff value of 0.5.

The α-helix, β-strand, and random coils are characteristics of secondary structural features that were assessed by applying various systems, including PSIPRED, SPOMA, and Phyre2. All three validation servers yielded concordant topological profiles, confirming identical boundary assignments for the alpha-helix, beta-strand, and coiled structural elements, although the distributions of amino acids between the most and least frequently identified forms differed dramatically. The PSIPRED program (Version 4.0) was mostly used for the final evaluation. **Figure 8** provides a graphic representation of the secondary structure’s characteristics. The PSIPRED server’s analysis shows that the MEV candidate contains 50.36% random coils, 26.05% β-strand, and 23.59% α-helix. Furthermore, the secondary structure analysis revealed that 2% of the regions were disordered (**Figure 9**).

### Modeling of 3D MEV candidate and validation

**Figure 10:**
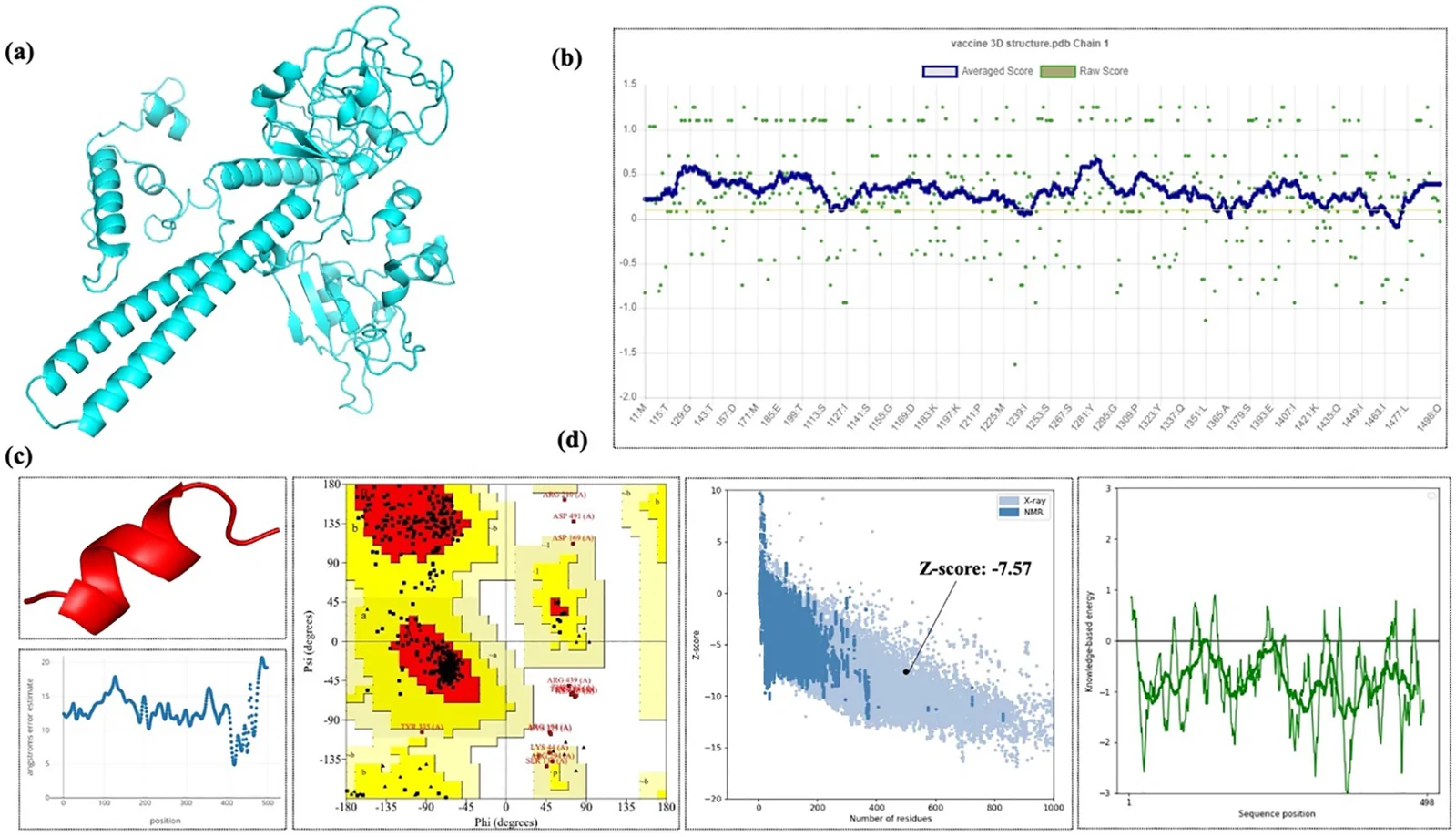
Structural refinement and *in silico* validation of the finalized MEV candidate. **(A)** 3D structural optimization was performed using the GalaxyRefine server to generate a refined 3D model for subsequent structural analyses. **(B)** ERRAT validation with overall quality factor of 79.53 was identified. ProSA-web energy profile evaluation verifying the model’s architectural validity through Z-score distribution and local quality indicators. **(C)** 3D representation of Gb-1 peptide in red color. **(D)** Ramachandran plot analysis demonstrating excellent backbone stereochemical quality post-refinement.

RoseTTAFold was utilized to predict the initial 3D structure of the engineered MEV candidate (**Figure 10A**). Following conformation optimization via the GalaxyRefine suite, Model 1 was prioritized over the other four generated structural candidates after demonstrating peak structural stability and refinement scores. The modified structure demonstrated structural stability with a Root Mean Square Deviation (RMSD) of 0.302 Å and a Global Distance Test-High Accuracy (GDT-HA) score of 0.8483 (numbers < 1.2 are generally regarded good). The modified model’s improved stereochemical quality was further validated by the MolProbity score of 2.126, which is in line with superior structural models. After comparative modeling using the native S protein, one of the projected models was selected using a confidence score of 0.02. ProSA-web, ERRAT, and Ramachandran plot evaluations were carried out to choose the optimal model (**Figure 10B**). ProSA-web was used to assess the quality of the optimal model and probable faults in the refined structure. Following model refinement, the Z-score was determined to be − 7.57, supporting the range typically observed in native proteins of comparable size. After the structure underwent additional ERRAT validation, an overall quality factor of 79.53 was identified. Gb-1 peptide 3D representation shown in **Figure 10C**. Furthermore, a Ramachandran plot analysis was performed, which showed that the favored, allowed, and outlier regions contained 85.9%, 10.8%, and 2.2% of the residues in the basic model (**Figure 10D**). As a result, the simulated MEV can be used for subsequent procedures like immunological simulation, MD simulation, and molecular docking.

### MEV disulfide bond engineering design

**Figure 11:**
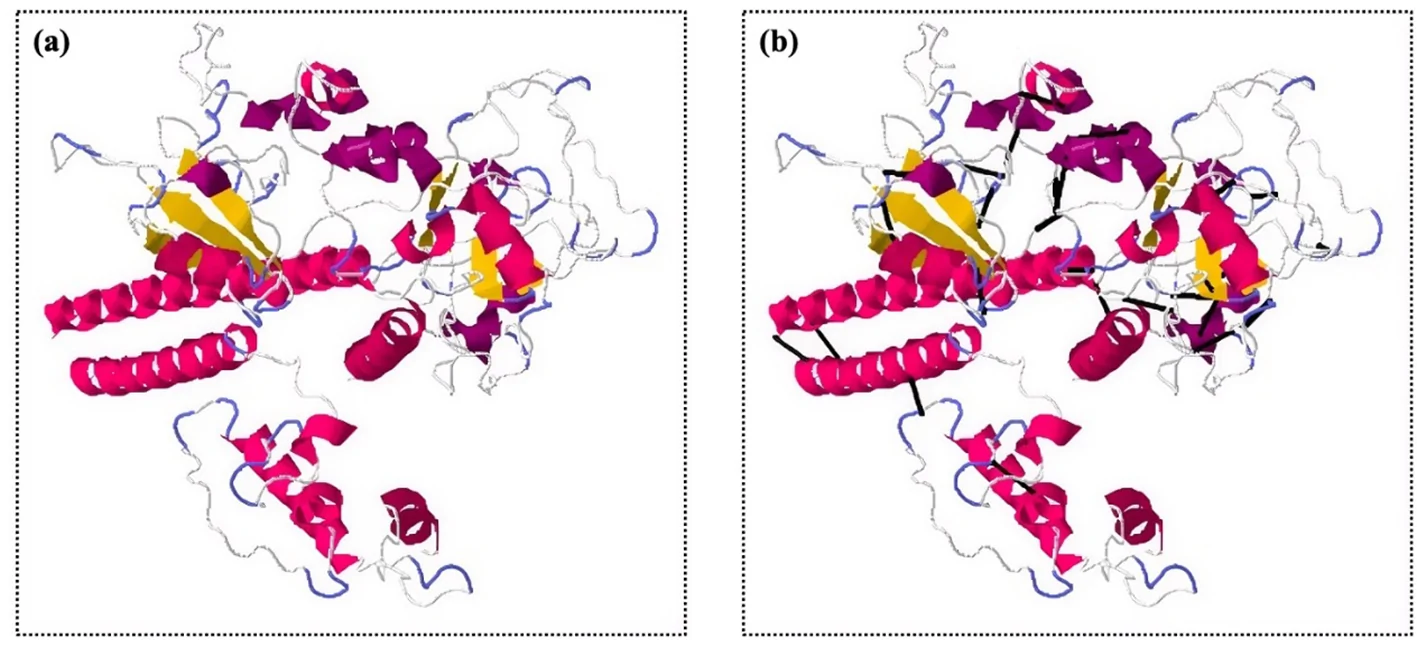
Computational stabilization of the vaccine architecture via engineered disulfide bonds. **(A)** Baseline visualization of the wild-type MEV candidate prior to mutation. **(B)** Selection of high-probability residue pairs for cross-linking. The CYS10-PRO338 alignment achieved the lowest overall conformational energy change (0.85 kcal/mol) with an optimal χ3 dihedral angle of +94.96°serving as an efficient single-substitution candidate (P338C) due to the native cysteine at residue 10. Furthermore, the pairs TYR227-GLY273 and LEU81-PRO219 emerged as viable candidates, presenting favorable energy values of 1.02 kcal/mol and 1.05 kcal/mol, with corresponding χ3 torsional angles of −92.73° and −84.40°.

Using the Disulfide by Design v2.12 server, 32 potential pairs of amino acid residues were identified as potential candidates for disulfide engineering. These couples were subsequently assessed using their energy score and χ3 angle. Three residue pairs in all satisfied the stringent structural and energy requirements needed for stable disulfide bond formation after a thorough investigation. The pair CYS10-PRO338 exhibited the lowest overall structural strain with an engineered bond energy of 0.85 kcal/mol and a highly favorable χ3 dihedral angle of +94.96°. Because position 10 natively harbors a cysteine residue, this site represents a highly efficient single-point mutation candidate (P338C). The pairs TYR227-GLY273 and LEU81–PRO219 also demonstrated strong engineering potential, yielding low energy values (1.02 kcal/mol and 1.05 kcal/mol, respectively) and near-ideal χ3 torsional profiles (−92.73° and −84.40°). Residue pairs characterized by high energy scores (2.2 kcal/mol) were systematically excluded from consideration to mitigate the risk of local structural distortion or protein misfolding (**Figure 11**).

### MEV candidate molecular docking with TLRs

To trigger downstream immune pathways effectively, an engineered vaccine must form highly stable complexes with host immunological receptors. We assessed peptide-MHC binding dynamics via target-specific molecular docking, utilizing thermodynamic binding affinity values to rank individual epitope interactions. Given that TLR engagement is an essential prerequisite for robust immune activation and infection containment, characterizing vaccine-TLR cross-talk remained a central focus. For this phase, the structural blueprint was finalized by conjugating the Gb-1 tag to the C-terminal of the construct, followed by comprehensive molecular docking simulations against TLR-2, 3, 4, 5, 7, and 8. The ClusPro results were selected for in-depth examination and presentation after molecular docking was carried out by employing two distinct servers to cross-validate the predicted interactions. The docking data demonstrated strong and beneficial interactions between the vaccine design and the TLRs in each docking session. The HADDOCK and ClusPro 2.0 servers were used to molecularly dock the vaccine’s improved 3D structure with TLRs. Complexes with the highest ranks were selected based on the MEV complexes from HADDOCK, accounting for the lowest mean RMSD values. Among the evaluated receptors, the MEV-TLR4 complex exhibited the lowest (most favorable) ClusPro weighted energy score. However, these weighted energy scores are computational ranking metrics used to compare predicted docking poses and should not be interpreted as experimentally determined binding free energies, receptor activation, or biological efficacy. The best docking findings were selected, as confirmed by additional verification of the HADDOCK data using the Pdbsum and LigPlot^+^ programs.

**Figure 12:**
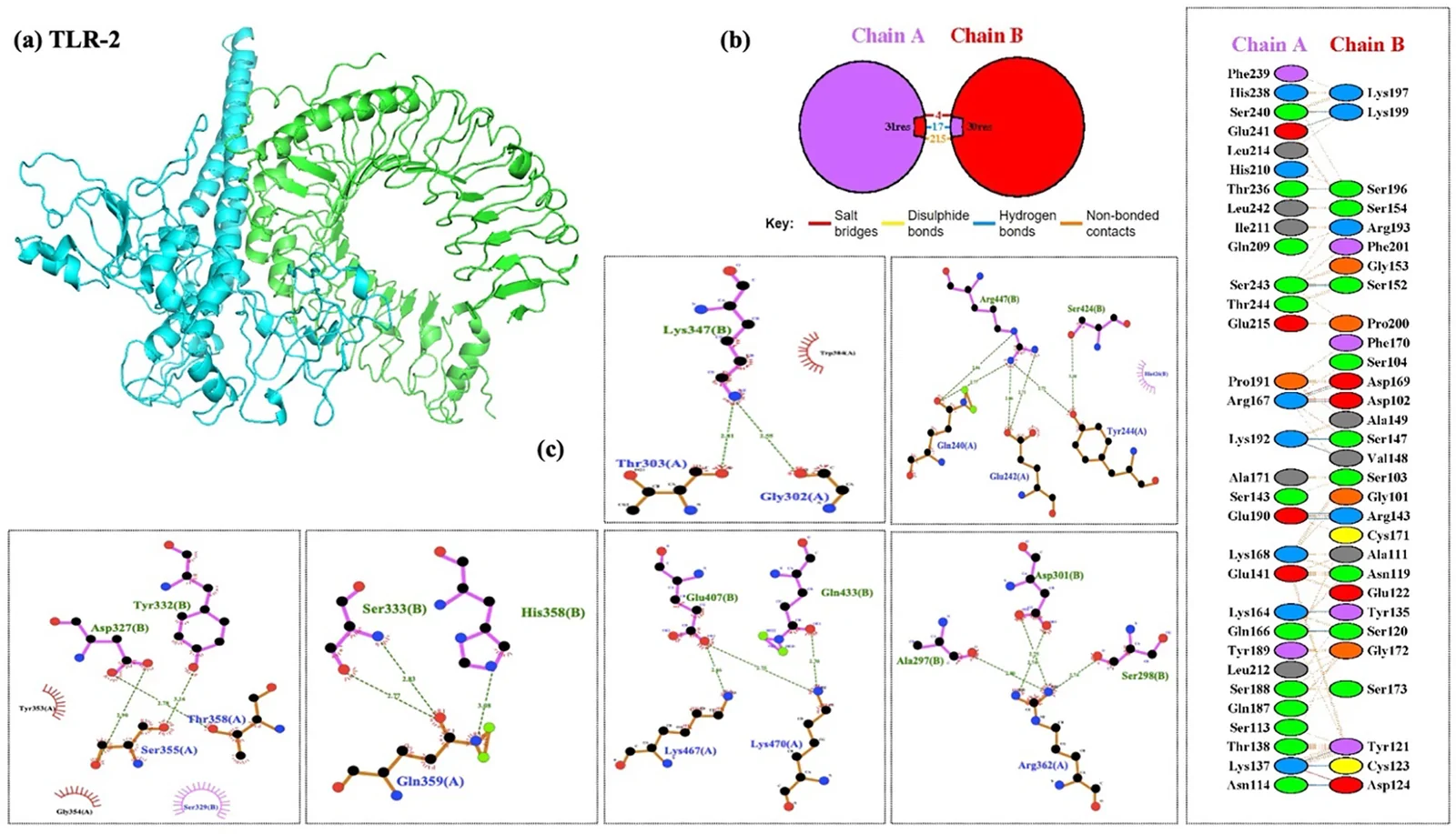
Molecular docking analysis of the MEV candidate complexed with human TLR-2. **(A)** 3D structural model showing the binding interaction between TLR-2 (green) and the vaccine candidate (cyan). **(B)** Overview of interface statistics detailing the number of salt bridges, hydrogen bonds, and non-bonded contacts linking Chain A and Chain **(B, C)** 2D schematic diagrams zooming into specific residue-to-residue bonding interactions across the docking interface.

**Figure 13:**
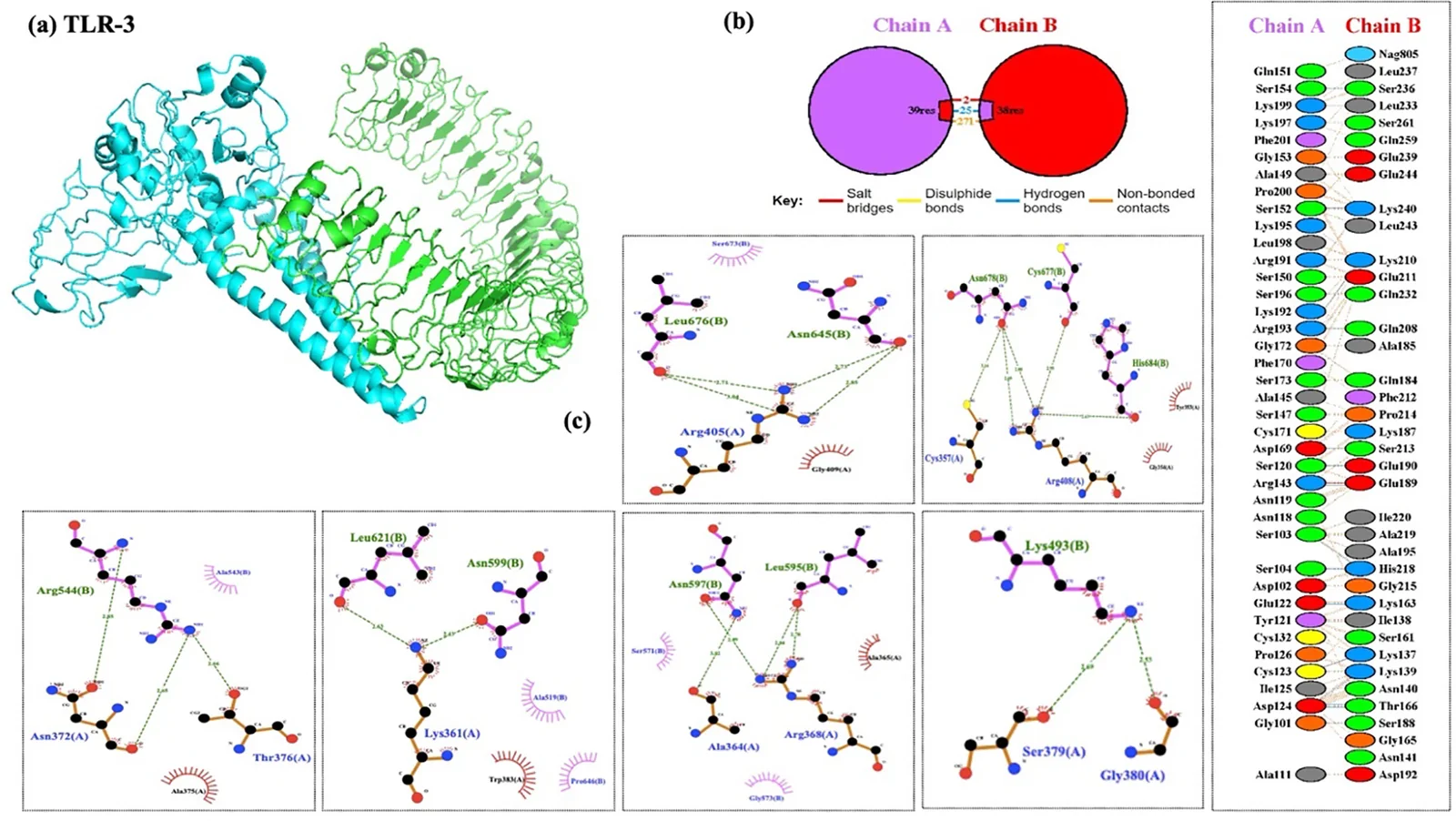
Intermolecular docking profile of the vaccine design and TLR-3 receptor. **(A)** Tertiary structure visualization of the docked complex framework. **(B)** Schematic breakdown of the binding boundary parameters between interacting chains. **(C)** Detailed PDBsum-derived interaction map illustrating the localized hydrogen bonds, salt bridges, and hydrophobic contacts stabilizing the complex interface.

**Figure 14:**
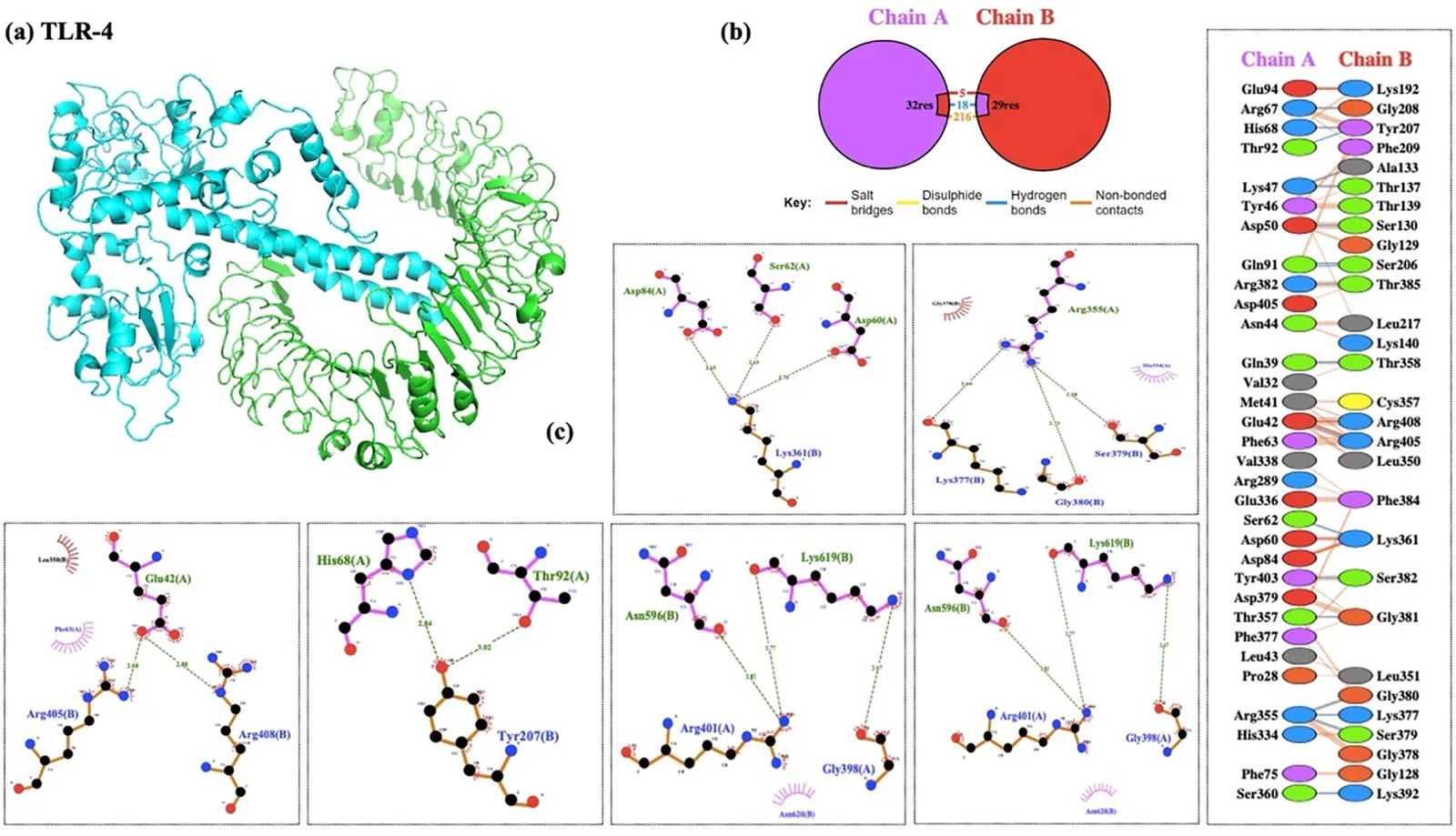
Interaction interface mapping between the engineered vaccine and TLR-4. **(A)** 3D spatial orientation of the docked receptor-ligand complex. **(B)** Macromolecular contact summary highlighting bond frequencies. **(C)** Specific 2D residue linkages defining the binding affinity and stabilizing network of the interface.

**Figure 15:**
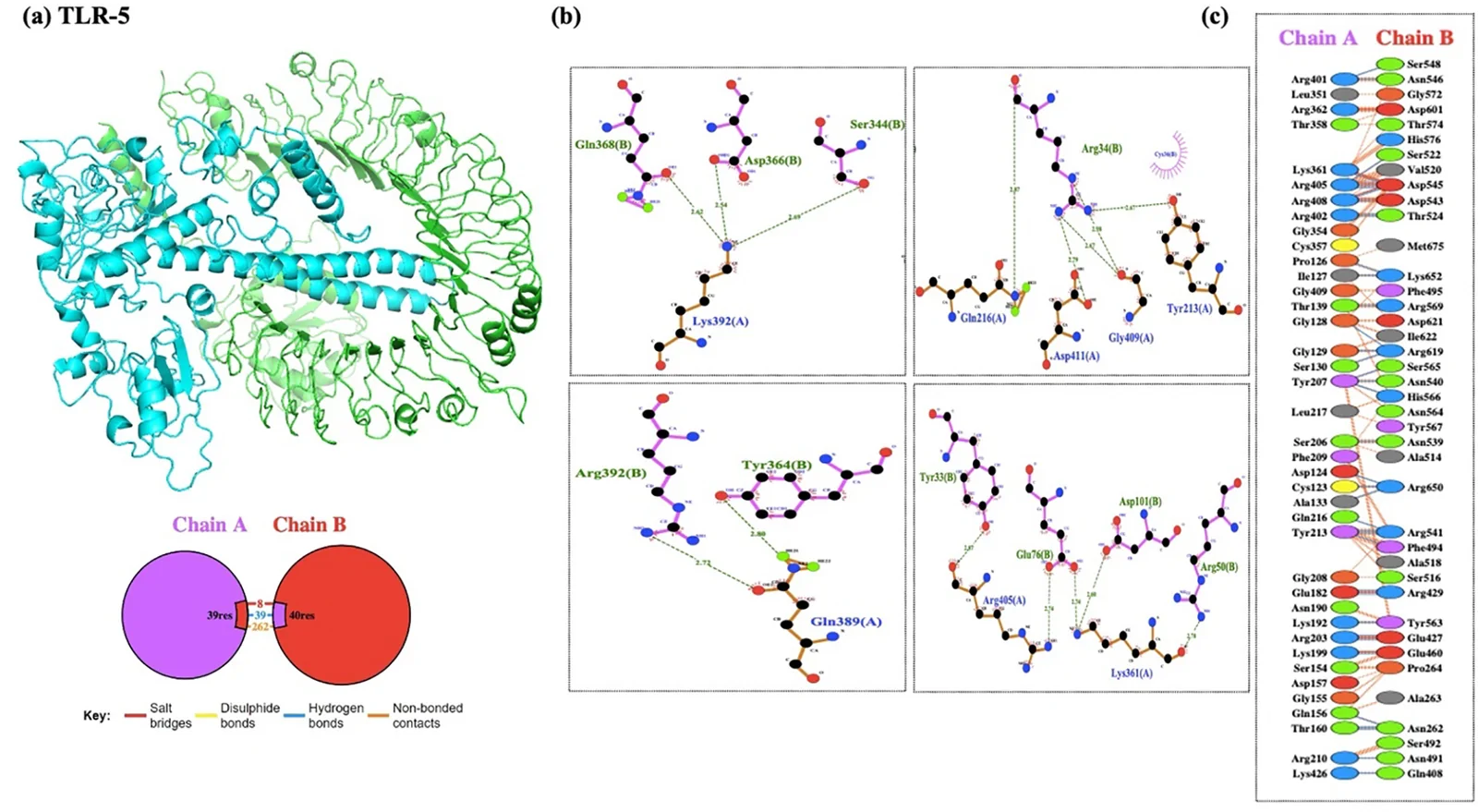
Molecular docking analysis of the MEV candidate complexed with human TLR-5. **(A)** 3D ribbon representation showing the structural binding interface between TLR-5 (green) and the vaccine candidate (cyan), supplemented by a macromolecular interaction summary. **(B)** Detailed 2D schematic diagrams highlighting specific residue-to-residue bonding networks within the binding pocket. **(C)** PDBsum layout mapping the full continuous network of hydrogen bonds, salt bridges, and non-bonded contacts between Chain A and Chain B.

**Figure 16:**
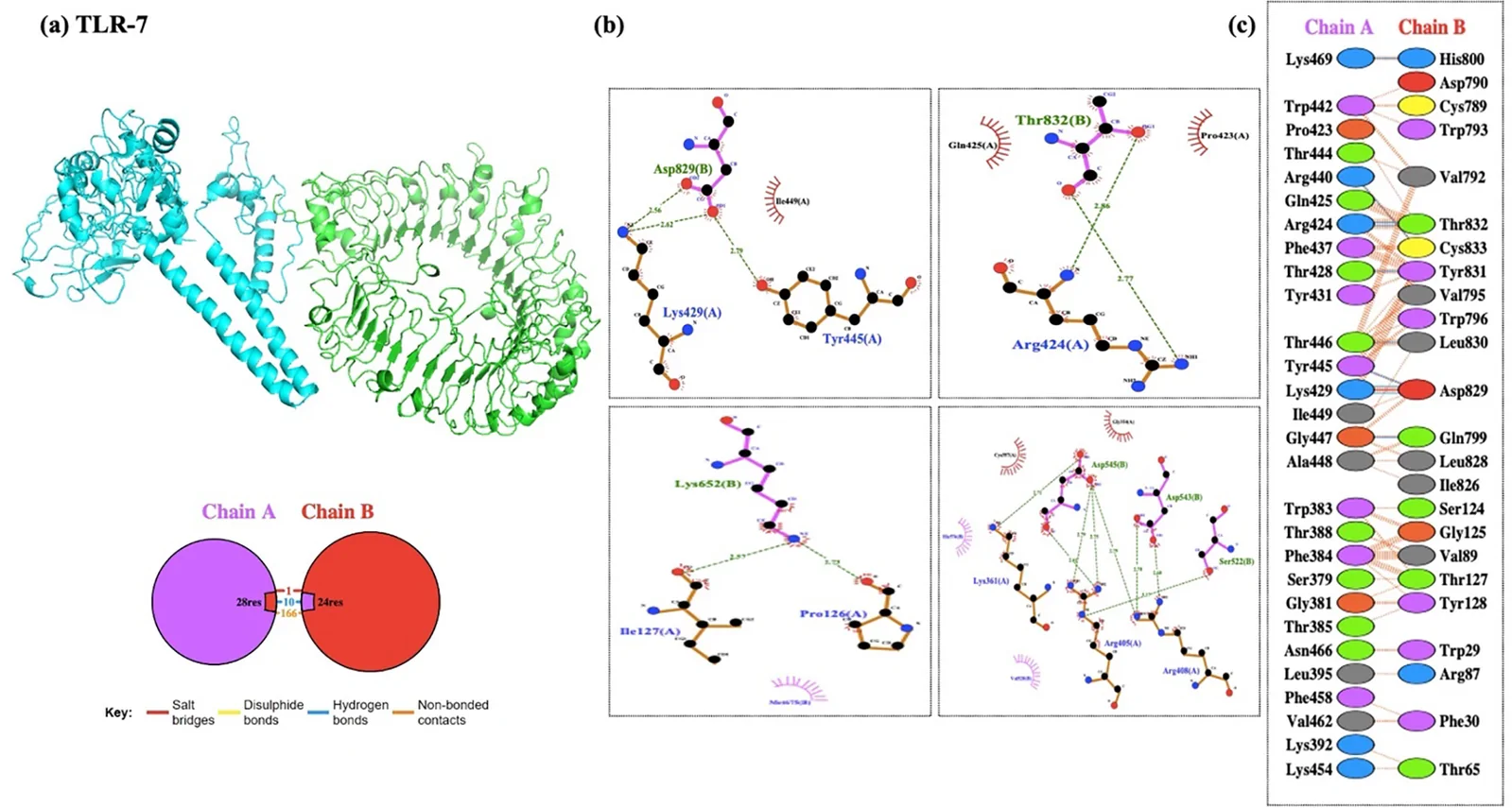
Intermolecular binding profile of the vaccine candidate paired with the TLR-7 receptor. **(A)** Tertiary configuration of the docked complex interface supplemented by macromolecular interaction statistics. **(B)** 2D schematic snapshots highlighting key localized atomic interactions. **(C)** Full linear layout mapping the precise amino acid bonds and hydrophobic contacts stabilizing the interface between Chain A and Chain B.

**Figure 17:**
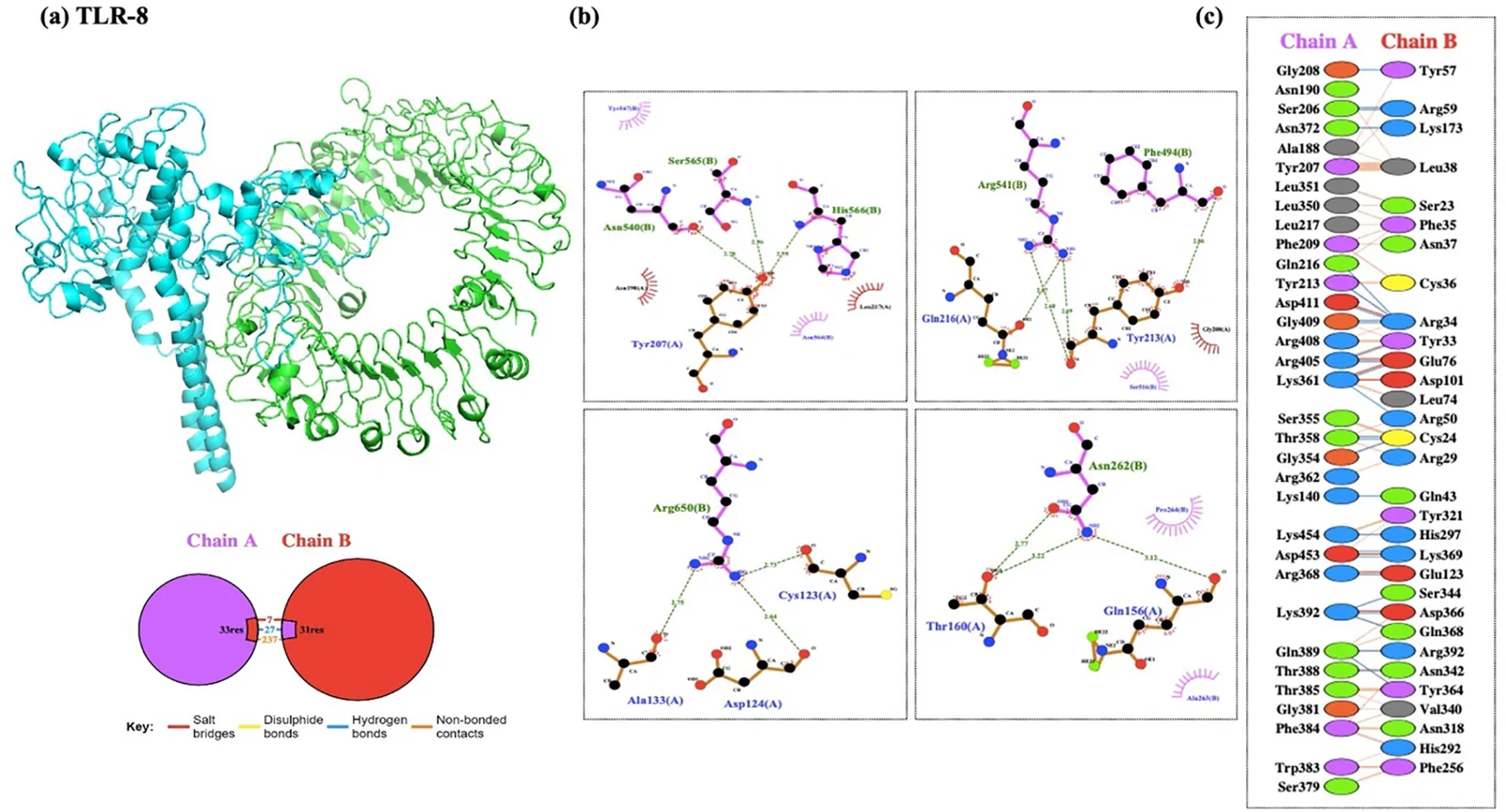
Interaction interface mapping between the engineered vaccine and TLR-8. **(A)** 3D spatial presentation of the docked complex structure. **(B)** Individual residue interaction panels detailing targeted hydrogen bonding trends. **(C)** PDBsum-derived layout illustrating the full network of chemical bonds and contact residues balancing the complex.

**Figure 18:**
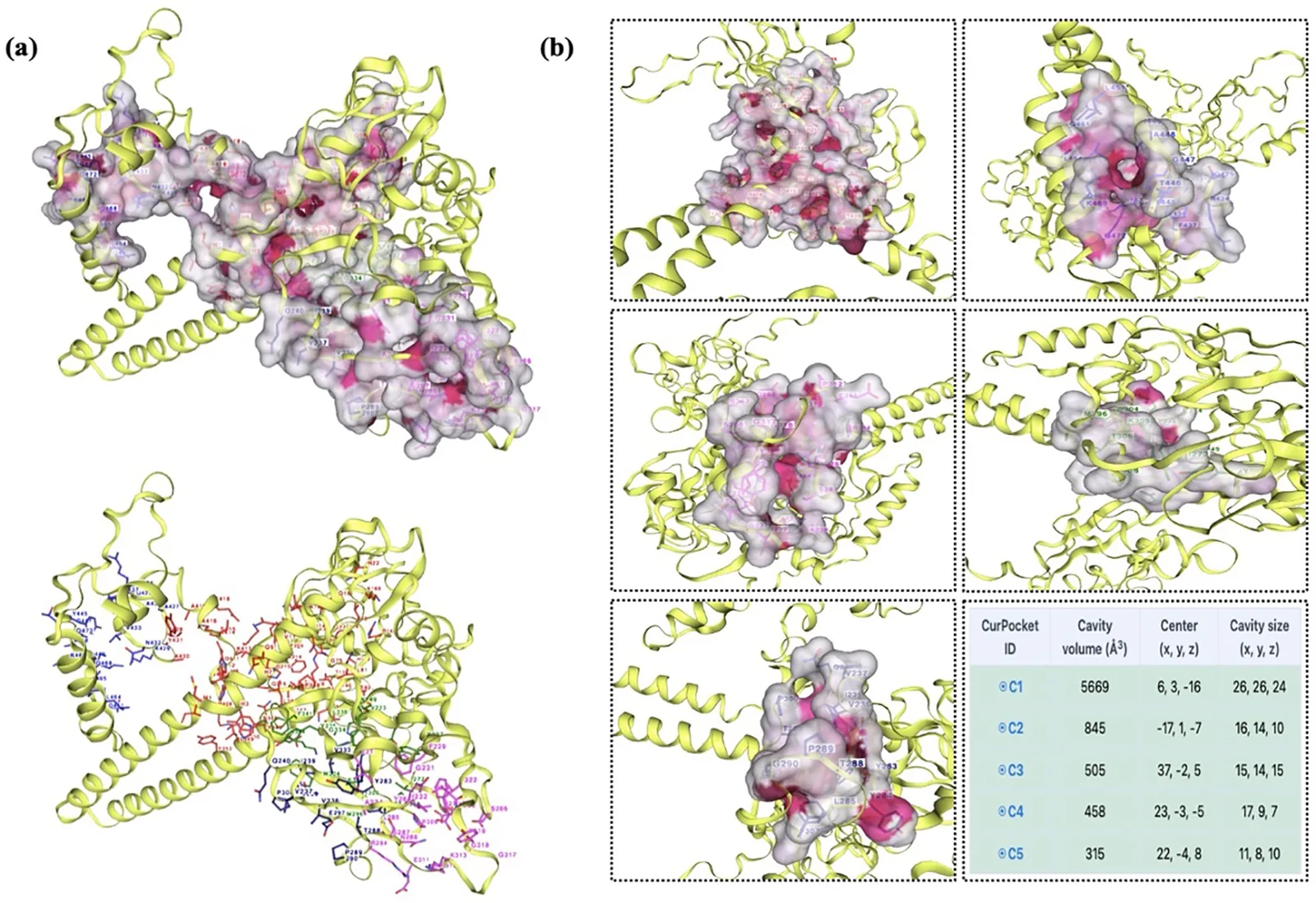
Topographical mapping of predicted binding cavities on the target protein utilizing CB-Dock2. **(A)** 3D structural rendering highlighting the five top-ranked binding pockets (C1-C5). Each cavity is labeled and color-coded according to its total volume profile. **(B)** Quantitative analysis of the pocket geometries. The detected cavities are ranked by size, spanning from the largest volume in cavity C1 (5669A°3) to the smallest in cavity C5 (315A°3), supplemented by their precise spatial center coordinates (x, y, and z) and dimensions. Due to their expanded volumes and optimal diameters, pockets C1, C2 and C3 present the highest probability for ligand accommodation, serving as the primary candidate sites for subsequent molecular docking.

The docking analyses performed using ClusPro provided a comparative assessment of the predicted interactions between the MEV candidate and the selected TLRs. Among the generated docking poses, the top-ranked complexes were selected based on their ClusPro weighted energy scores. The predicted weighted energy scores for the MEV complexes with TLR2, TLR3, TLR4, TLR5, TLR7, and TLR8 were −1205.4, −1312.1, −1531.7, −1240.2, −1145.2, and −1416.1, respectively. Among the evaluated receptors, the MEV–TLR4 complex exhibited the lowest (most favorable) ClusPro weighted energy score. These scores were used solely to rank and compare the predicted docking poses and should not be interpreted as experimentally determined binding free energies or direct measures of biological binding affinity (**Figures 12**–**17**). The predicted docking results suggest favorable interactions between the MEV construct and the evaluated TLRs. To further characterize the potential binding interfaces, the CB-Dock2 server was employed to identify putative binding cavities within the receptor structures. Five distinct binding cavities were identified, each differing in volume, location, and spatial dimensions, providing potential binding sites for docking analysis (**Figure 18**). These structural features offer additional insights into the predicted receptor binding pockets and may facilitate subsequent computational docking and structure-based design studies. Overall, the computational analyses indicate that the MEV is predicted to interact with the evaluated TLRs; however, the biological significance and potential receptor activation associated with these interactions require experimental validation.

### MD simulation

**Figure 19:**
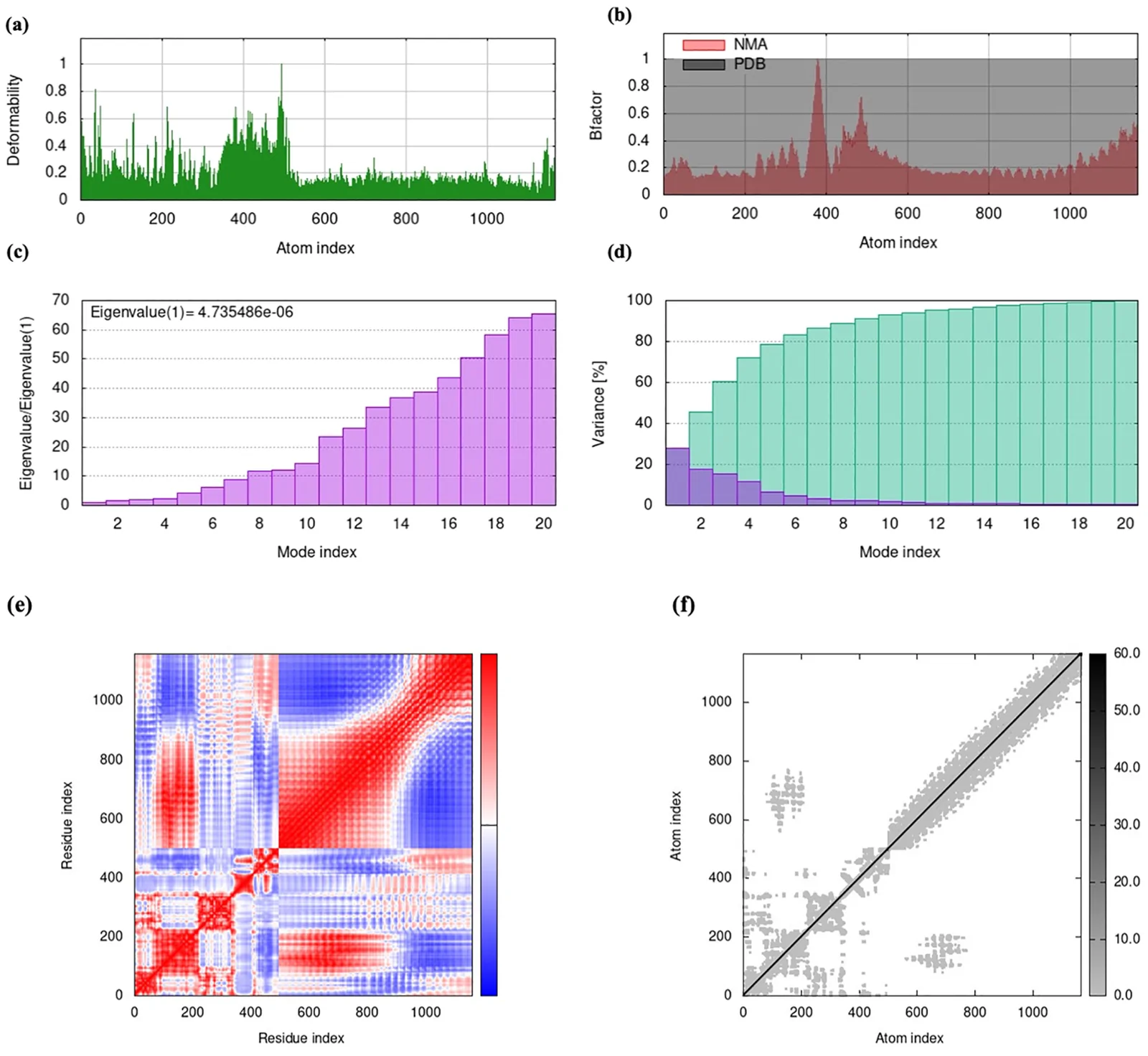
Computational stability and deformability analysis of the vaccine-TLR-4 docked complex. **(A)** Deformability simulation tracking flexible hinge points within the primary chain. **(B)** Evaluation of atomic variance metrics represented through NMA-derived B-factor calculations. **(C)** The characteristic eigenvalue of the docked system, reflecting its overall structural resistance to distortion. **(D)** Color-coded covariance matrix illustrating the spatial motion correlations across distinct residue coordinates. **(E, F)** An elastic network framework modeling atomic connections as localized springs, where darker gray tones distinguish stiffer structural regions.

NMA was performed using the iMODS server to computationally assess the predicted structural flexibility and stability of the designed MEV candidate-TLR4 complex. Unlike explicit molecular dynamics simulations, iMODS evaluates the intrinsic collective motions of the protein complex based on normal mode analysis, providing insights into its conformational mobility and mechanical stability. The analysis generated deformability plots, B-factor profiles, eigenvalues, covariance matrices, and elastic network models to characterize the dynamic behavior of the complex. The relatively high eigenvalue suggested that greater energy would be required to induce conformational deformation, indicating favorable predicted structural stability of the MEV candidate-TLR4 complex. Furthermore, the covariance matrix and elastic network analyses revealed coordinated residue motions and a stable interaction network within the complex. Overall, these computational findings suggest that the designed vaccine-receptor complex possesses favorable predicted structural stability and conformational flexibility; however, these observations are based solely on normal mode analysis and require validation through experimental studies or explicit molecular dynamics simulations. The main-chain deformability investigation highlighted the hinges of the structure by identifying significantly flexible places (**Figure 19A**). The NMA-derived β-factor scores indicate the degree of thermal atomic displacement, pinpointing the localized mobility and positional uncertainty of each atom within the macromolecule (**Figure 19B**). The complex was found to have an eigenvalue of 4.735486 × 10−6, which is associated with the energy required for structural distortion (**Figure 19C**). The covariance matrix illustrates the coordinated dynamics between residue pairs across the protein backbone, distinguishing between correlated (red), uncorrelated (white), and anti-correlated (blue) structural fluctuations (**Figure 19D**). The elastic network model was also used to depict the relationships between atoms and the forces operating on them (**Figures 19E, F**). The results of the MD simulation were used to validate the stability of our vaccine model, and the graph shows that the majority of the active site residues stayed stable.

### Codon optimization and in silico cloning

**Figure 20:**
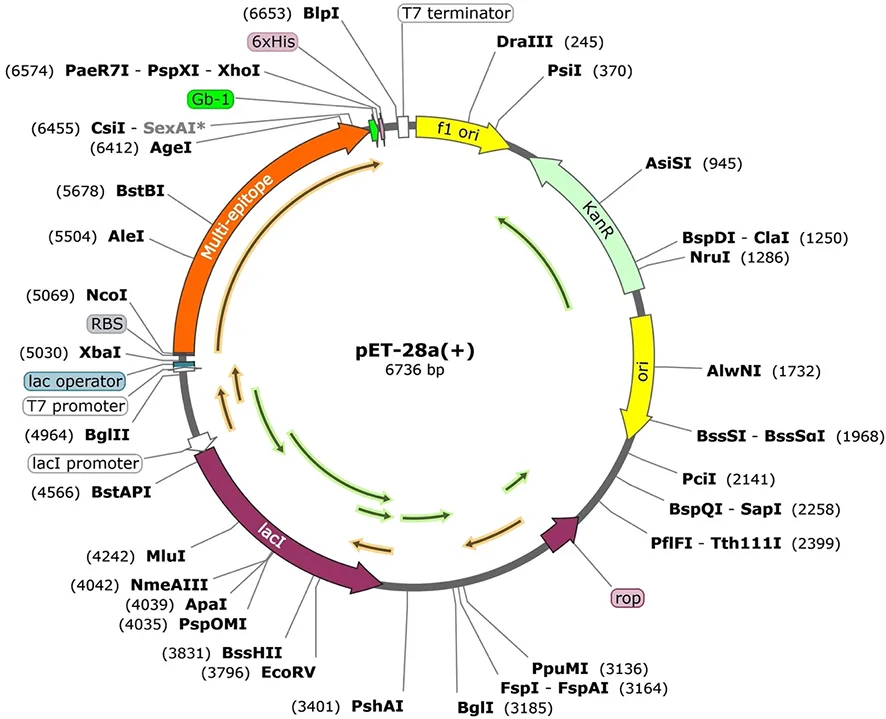
In silico restriction cloning scheme of MEV candidate into the pET28a(+) expression vector using SnapGene software. The vaccine-encoding nucleotide sequence is represented by a red segment, while the Gb-1 sequence is marked as a green line. The unique restriction sites for *NcoI* and *XhoI* are incorporated at the 3’- and 5’-terminals boundaries of the insert, respectively. Additionally, the N-terminal His-tag native to the pET28a vector framework is highlighted by a purple box to facilitate downstream purification.

**Figure 21:**
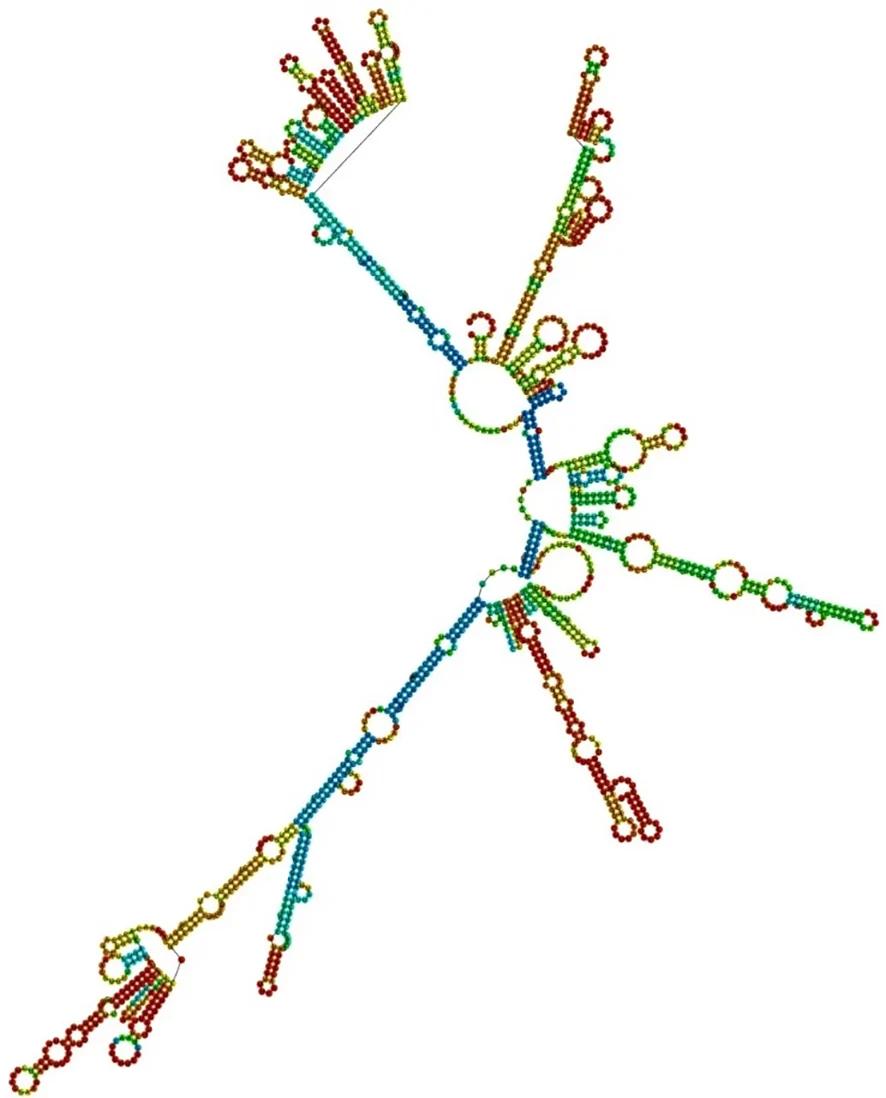
Conformational analysis of the MEV candidate mRNA transcript. The RNAfold server was deployed to map the secondary folding pattern of the MEV sequence. The predicted mRNA secondary structure exhibited a minimum free energy (MFE) of −499.28 kcal/mol, indicating a thermodynamically favorable conformation according to computational prediction.

The Java Codon Adaptation Tool was utilized to optimize the vaccine’s nucleotide sequence for robust expression in Escherichia coli. Modifying the software parameters allowed the systematic exclusion of unintended bacterial ribosome binding sites, rho-independent terminators, and restriction enzyme sites from the synthetic cDNA sequence. This back-translated construct was cloned *in silico* into the *E. coli* expression system to maximize potential protein yield. Following optimization, the sequence demonstrated an excellent Codon Adaptation Index value of 0.975, indicating optimal translational efficiency. The MEV candidate GC content of 51.27% indicates that the vaccination protein is highly expressed in the *E. coli* K12 expression system. *NcoI* and *XhoI* enzymes were selected, restriction sites were added to the 5′ and 3′ ends of the sequence, and a stop codon was placed in accordance with the Gb-1 sequence in order to clone the vaccine gene for expression in *E. coli* onto the pET-28a (+) plasmid (**Figure 20**). The vaccine’s mRNA structure was examined using RNAfold, and the minimal free energy (MFE) score was −499.28 kcal/mol (**Figure 21**). The stability of the vaccine after *in vivo* expression depends on improved mRNA stability, which is indicated by a lower MFE score. These results show that the MEV candidate is a viable and economical strategy for SARS-CoV-2 prevention. To further validate these findings further experimental research is necessary.

### MEV candidate immune simulations

**Figure 22:**
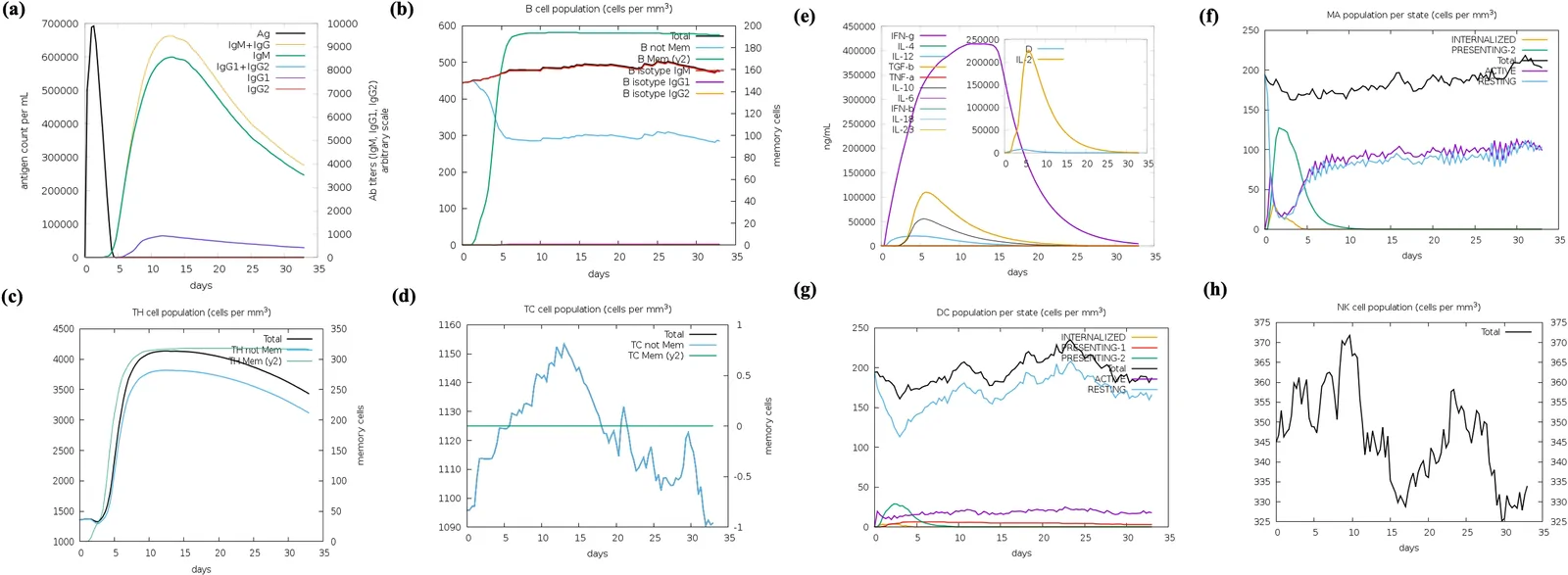
Computational immune simulation profile of the formulated vaccine candidate executed via the C-ImmSim server. **(A)** Kinetics of primary immunoglobulin and B cell population expression. **(C, D)** Robust activation of TH and TC cell population, characterized by strong immunological memory and rapid antigen clearing, as evidenced by the sustained proliferation of T cells. **(E, F)** Elevated expression levels of key cytokines, including IFN-γ, IL-23, IL-10, IL-8, and IL-6, validating the construct’s ability to trigger an antiviral inflammatory defense network. **(G, H)** Proliferative expansion of DCs and NK cells signaling enhanced antigen presentation by APCs.

To evaluate the potential immune response elicited by the designed vaccine construct, an in silico immune simulation was performed using the C-ImmSim server. This computational analysis was conducted to predict the temporal dynamics of the host immune response following the simulated vaccine administration. The simulation predicted increased populations of CTLs, HTLs, B cells, and NK cells, accompanied by elevated levels of selected cytokines, including interleukins and interferons, suggesting the potential induction of both cellular and humoral immune responses. Furthermore, the simulated primary and secondary immunization phases demonstrated an initial increase in combined IgM and IgG antibody levels, followed by elevated IgM, IgG1 + IgG2, and IgG1 responses. The simulation also predicted a progressive decline in antigen levels following successive immunizations, consistent with an enhanced simulated adaptive immune response (**Figure 22A**). Additionally, the simulated persistence of memory HTL, CTL, and B-cell populations suggested the potential induction of immunological memory, accompanied by predicted immunoglobulin class switching and sustained antibody responses during the simulated vaccination period (**Figures 22B–D**). The immune simulation also predicted increased levels of selected cytokines, including IL-2 and IFN-γ (**Figure 22E**). Furthermore, the simulation indicated the recruitment and persistence of dendritic cells (DCs) and macrophages during the simulated antigen presentation process (**Figures 22F, G**). An increase in the simulated NK cell population was also observed following the simulated vaccine administrations (**Figure 22H**). Collectively, these computational predictions suggest the potential of MEV candidate to elicit both innate and adaptive immune responses following the simulated immunization schedule. However, these findings represent in silico predictions and require experimental validation.

## Discussion

The World Health Organization (WHO) has proclaimed SARS-CoV-2 to be a global pandemic that affects people of all ages. WHO declaration of COVID-19 as a worldwide public health emergency has prompted scientists to create treatments, including potential medications and vaccines, to combat the illness (52). Traditional vaccine production techniques typically deploy complete organisms or large protein antigens. This approach often results in an redundant antigenic load, which increases the likelihood of triggering allergic or hyper-inflammatory patient responses. Computational immunoinformatic approaches offer a streamlined alternative to traditional screening, allowing researchers to efficiently identify the optimal antigenic epitopes needed to formulate MEV candidates (53). Compared to full-protein immunization strategies, MEVs screen viral genomes to select only the most immunogenic peptide fragments. This design elicits a highly specific protective response while avoiding any potential restoration of viral pathogenicity (54).

The selection of S and N proteins was based on their complementary immunological roles in SARS-CoV-2 infection. The S protein mediates viral attachment and entry into host cells and is the principal target of neutralizing antibodies, making it the foundation of most current vaccine platforms. However, its high mutation rate, particularly among emerging variants, can compromise antibody-mediated protection (13). In contrast, the N protein is highly conserved across SARS-CoV-2 variants and contains numerous immunogenic T-cell epitopes that contribute to durable cellular immunity (15). Therefore, the present study focused on combining selected epitopes from both proteins to broaden immune recognition by integrating neutralizing antibody targets with conserved T-cell antigens. Although other structural, non-structural, and accessory proteins are involved in viral replication and pathogenesis, the S and N proteins remain the most extensively characterized and immunologically validated targets for epitope-based vaccine development. Accordingly, our objective was to develop a preventive MEV targeting these two proteins, which are critical determinants of viral entry, antigenicity, and protective immune responses (55). Previous multi-epitope vaccine research using epitopes from both the S and N proteins supports this approach. After developing a synthetic multi-epitope SARS-CoV-2 antigen with conserved and immunogenic epitopes derived from S and N proteins, Invençao et al. reported more than 90% global population coverage, which was followed by experimental evidence of CD4^+^ and CD8^+^ T-cell proliferation following DNA vaccine. In contrast, the current study uses an expanded computational approach that includes structural modeling, receptor docking, dynamic analyzes, and immune simulation in addition to integrating S- and N-derived epitopes in a comparable manner and adding the Gb-1 immunomodulatory component. These variations provide the complimentary justification for merging S-derived epitopes with conserved cellular immunological targets from N while employing further computational analyzes to describe the suggested construct (56).

The proposed MEV was designed using integrated immunoinformatics and molecular modeling approaches to identify and assemble highly immunogenic CTL, HTL, and B-cell epitopes capable of eliciting both humoral and cell-mediated immune responses (57, 58). Unlike full-length recombinant protein vaccines, the engineered construct restricts immune exposure to carefully selected immunodominant epitopes, thereby minimizing unnecessary antigenic burden while potentially reducing the risk of adverse allergic reactions (57). Furthermore, the multi-epitope framework enables simultaneous presentation of multiple MHC class I- and class II-restricted epitopes, promoting coordinated activation of diverse T-cell subsets and B-cell responses. The incorporation of an adjuvant further enhances immunogenicity and may prolong immune persistence, while the computational design strategy circumvents many limitations associated with conventional pathogen cultivation and empirical vaccine development (59). Collectively, these advantages have established MEV design as a rational and promising vaccine development strategy, with preclinical studies demonstrating its potential to induce broad, durable, and protective immune responses (60).

The predicted immunogenic epitopes identified from the SARS-CoV-2 S and nucleocapsid N proteins were assembled into MEV candidate using GSG and GSGG linker sequences. In addition, the Gb-1 (GP-2 binding) peptide, previously identified in our laboratory through a phage display screening approach, was incorporated at the C-terminus of the construct. Previous experimental studies demonstrated that Gb-1 possesses immunostimulatory properties and preferentially promotes a Th2-type immune response, providing the rationale for its inclusion in the present vaccine design (37). Accordingly, Gb-1 was incorporated as a potential immunomodulatory ligand with the aim of enhancing the predicted immunogenic properties of the MEV while preserving the integrity of the selected epitopes. However, the contribution of Gb-1 to the immunogenicity of the proposed MEV candidate remains a computational design hypothesis and requires experimental validation. The candidate B cell, HTL, and CTL epitopes were systematically evaluated using several computational safety and efficacy thresholds. To be selected, individual peptides had to prove antigenic and immunogenic, show broad-spectrum promiscuity toward numerous MHC Class I and II alleles, and maintain shared sequence overlapping regions between the T cell subsets. The epitopes must be highly conserved, non-toxic, antigenic, non-allergic, and capable of binding to MHC-I/II alleles. They should also overlap with CTL, HTL, and B cell epitopes. Based on the positive outcomes of the initial screening stage, 80 B cell and T cell epitopes were finally considered. After strict filtering rounds, only highly antigenic epitopes lacking toxic, allergenic, or human proteomic homologies were advanced for construct assembly. The selected HTL epitopes were further vetted for their ability to mediate IFN-γ, IL-4, and IL-10 cytokine responses. Habib et al. previously implemented a similar computational strategy, confirming the validity of this screening framework to design a highly immunogenic MEV model against HIV-1 (61).

Therefore, the ability to predict TAP transport and proteasomal cleavage is crucial for improving ME-based vaccines. All selected epitopes T cells epitopes seem to be available for immunological feedback during the antigen processing and presentation performed by capable APCs, according to the results from the NetCTL 1.2 server. Helper T cells are crucial for acquired or adaptive immunity since they assist the CTL in eliminating infected cells as well as the B cell in producing and releasing antibodies. They become effectors upon activation and presentation on the surface of APCs, maturing into a particular form of HTL needed for a particular immune feedback (62).

In this study, we assessed each identified HTL epitope’s ability to produce cytokines and IFN-γ. Based on their anticipated SVM scores, most of our HTL epitopes showed favorable stimulation of IFN-γ and cytokine production. By mobilizing macrophages and natural killer cells while simultaneously upregulating major histocompatibility complex antigen presentation pathways, the selected T cell epitopes amplified the capacity of interferon-gamma to orchestrate both innate and adaptive immune cascades (63).

Developing effective vaccine immunogens requires addressing both the hyper-variability of SARS-CoV-2 and the extreme polymorphism of human HLA types. In this study, we deliberately selected a heterogeneous pool of epitopes with broad allele binding profiles to optimize global demographic coverage. This multi-epitope targeting strategy yielded calculated population coverage metrics of 98.55% and 81.81% for the MHC Class I/II epitope sets, respectively. Furthermore, the MEV candidate demonstrated a notable cumulative population coverage, especially in regions where Omicron is highly prevalent. The Omicron group of SARS-CoV-2 subtypes had an epitope conservation score of 97.38% based on the size of the final defined epitopes. Although there are clear variations based on the antigenic regions and HLA alleles used, the estimated population coverage of the current construct is generally similar with other computational and experimental MEV investigations. A conserved MEV targeting epitope was developed by Rastogi et al. from the S, M, and E proteins of SARS-CoV-2 variants of concern (64). They also found stable interactions with TLR4, HLA class I, and HLA class II, as well as anticipated innate and adaptive immune responses. Similarly, Deepthi et al. showed that a spike-based MEV with CD4^+^ and CD8^+^ T-cell epitopes covered almost 90.03% of the world’s population (65). The ProtParam server predicted MW and pI of the MEV to be 55.47 kDa and 9.84, respectively. The theoretical pI of 9.84 indicates that the MEV candidate is basic (alkaline) in nature, providing a physicochemical characteristic relevant to its computational evaluation. The MEV candidate half-life was reported to be 30 hours *in vitro* in mammalian reticulocytes, whereas it was shown to be 20 hours and more than 10 hours *in vivo* in yeast and *E. coli*, respectively. In the same way, the protein’s hydrophilic nature and potential for better interactions with surrounding water molecules are shown by the negative GRAVY score (-0.851). The assessment of physicochemical characteristics in comparison to earlier research revealed the vaccine’s adequate solubility, which is crucial for boosting the immune response and bioavailability while lowering toxic effects and other adverse effects. When compared to the study by Safavi et al., the vaccine’s heat stability as determined by the aliphatic index was lower (66, 67). The current construct’s overall physicochemical profile is in line with features documented for prior computationally generated SARS-CoV-2 MEV candidates. A construct with optimal codon use for expression in *E. coli* K12 and good antigenicity, non-allergenicity, and non-toxicity was described by Deepthi et al. (65). A spike-based MEV with advantageous physicochemical characteristics, anticipated solubility, and structural resemblance to the natural protein was also reported by Samad et al. (68). These results support the use of computational physicochemical screening as a first step in vaccine design optimization and are generally in line with current forecasts.

The RoseTTAFold online tool used the comparative modeling approach to estimate the candidate MEV’s 3D model. Following comparison modeling using the native S and N proteins as a template, one of the predicted models was selected using a confidence score of 0.02. ProSA-web, ERRAT, and Ramachandran plot evaluations were carried out to choose the optimal model. According to this research, the vaccine should work as a vaccine because it has a structurally suitable shape. Interestingly, the analysis’s selected locations showed the largest content of amino acids (69). Additionally, the optimal MEV model was docked against several TLRs following synthesis and refinement. The computational data indicated robust binding affinities and low binding energy scores between the finalized MEV candidate and the selected TLRs. Furthermore, cross-interface contact map analysis exposed highly individualistic patterns of interchain residue-to-residue contacts within the respective receptor-vaccine complexes. Previous studies have implicated TLR4 in the pathogenesis of COVID-19, with evidence suggesting that the SARS-CoV-2 S protein can interact with TLR4 and contribute to the activation of innate immune signaling pathways. Moreover, inhibition of TLR4-mediated signaling has been reported to attenuate inflammatory responses and reduce the severity of SARS-CoV-2 infection in experimental models, supporting its relevance as a biologically meaningful receptor for computational interaction studies. This suggests that the virus may be binding to and activating TLR4 to boost the production of ACE2, which facilitates viral entry (70). Numerous studies have emphasized the significance of how vaccines interact with TLR4. Totura et al. showed that TLR4-deficient mice are more susceptible to SARS-CoV-2 infection than wild-type mice (71). Similar to this, Hu et al. found that exposure to SARS-CoV-2 infection increased TLR4 expression, indicating the significance of TLR in immune response stimulation (72). Analysis of the docking interfaces identified distinct interaction patterns between the MEV candidate and the evaluated TLRs. In the predicted TLR3 and TLR8 complexes, three domain regions contributed prominently to the intermolecular contacts. The MEV candidate exhibited relatively extensive residue interactions with TLR7 and TLR8, whereas more localized interaction interfaces were observed for the TLR2 and TLR3 complexes. Collectively, these docking results suggest differential predicted binding modes between the MEV candidate and the selected TLRs. However, these computational findings represent potential molecular interactions only and should not be interpreted as direct evidence of receptor activation or downstream immune signaling, which require experimental validation.

NMA using the iMODS server was used to computationally assess the intrinsic flexibility and predicted structural stability of the MEV-TLR4 complex. Unlike explicit molecular dynamics simulations, NMA evaluates the collective motions of biomolecular complexes and provides insights into their potential conformational behavior. The relatively high eigenvalue obtained from the iMODS analysis suggests that greater energy would be required to induce structural deformation, indicating favorable predicted structural stability of the complex. Moreover, the deformability profile and covariance matrix revealed coordinated residue motions and limited flexible regions, supporting the overall structural integrity of the vaccine-receptor complex. Although these computational findings provide valuable insights into the dynamic properties of the proposed MEV candidate, they represent predictive analyses and should be validated through explicit molecular dynamics simulations and experimental studies. We were able to more precisely investigate the stability and conformational changes in the vaccine-receptor complex by analyzing a number of descriptors that were obtained from the trajectory data of MD simulations (73). Consequently, these trajectories clarify the molecular flexibility and specific structural characteristics defining the vaccine-TLR interface. Recent computational investigations also support the projected structural stability of the vaccine-receptor combination. While Deepthi et al. and Mohammadipour et al. similarly found favorable dynamic stability of their vaccine-TLR complexes, Rastogi et al. used molecular dynamics and normal mode analysis to assess the stability of their MEV-receptor complexes (64, 65, 74). The computational evaluation of vaccine-receptor interactions is strengthened by the employment of complementing structural and dynamic analyzes in previous investigations and the current work.

The designed construct was cloned *in silico* to achieve maximal expression in the *E. coli* expression system. Protein expression is influenced by a number of variables, including GC concentration and CAI. A higher likelihood of protein expression is indicated by a CAI score greater than 0.8, which displays the degree of codon use in an organism. For effective protein production, any gene’s GC content should range from 30% to 70% (75). The GC and CAI contents in this work were 51.27% and 0.975, respectively. Finally, the optimized sequence was *in silico* cloned into a pET28a(+) vector using SnapGene and the restriction enzymes *XhoI* and *NcoI*. This vector is an excellent tool for producing high quantities of the desired peptide since it contains a strong T7 promoter. Furthermore, it possesses suitable restriction enzyme sites (76). Additionally, the secondary structure of the MEV candidate mRNA was examined using the RNAfold (version 2.1.0) program. A more stable vaccine within the body was indicated by the software’s minimal free energy value of 499.28 kcal/mol. This stability is important while developing a vaccine.

The immune response simulation of the vaccine design revealed elevated levels of IL-2 and IFN-γ. Immune response simulation results show that IgM immunoglobulin has a higher level than IgG1 and IgG2, as well as the IgG1+IgG2 response, with the highest level occurring during the first two weeks. The antigen concentration dropped as a result of this rise in the antibody titer. The HTL response results showed that memory helper cell and active T-helper cell levels were high during the simulation period, and the population of active T-cytotoxic cells was elevated. Our results are consistent with the research conducted by Safavi et al., who shown that their vaccine might boost cellular and humoral immunity (77).

Targeting a highly conserved viral antigen that can elicit extensive T-cell responses is one benefit of including the SARS-CoV-2 N protein, but its inclusion also needs to be carefully considered. Theoretical concerns about disease promotion have been raised by prior research suggesting that particular N protein-specific immune responses may contribute to excessive inflammation or immunopathology (15). Instead of using the entire N protein in the vaccine design, the current study only used computationally selected N-derived epitopes that were anticipated to have positive antigenicity, non-allergenicity, and non-toxicity. Although the goal of this epitope-based approach was to reduce the likelihood of off-target immune reactions, unexpected immunological consequences cannot be ruled out by computational predictions alone. Therefore, thorough *in vitro* and *in vivo* research will be necessary to assess the safety, immunogenicity, and possible inflammatory effects of including N-derived epitopes into the suggested vaccine candidate. Although immunoinformatics approaches provide valuable strategies for rational vaccine design, several biological aspects remain beyond the predictive capacity of computational analyses. Potential factors including immune tolerance, epitope masking caused by structural arrangement, and excessive activation of innate immune pathways require experimental investigation. In particular, predicted interactions between vaccine constructs and immune receptors should not be interpreted as direct evidence of immune activation or protective efficacy. Therefore, comprehensive *in vitro* and *in vivo* evaluations are essential to determine the immunogenicity, safety, and protective potential of the proposed MEV candidate (78).

Compared with previously reported computational MEV studies targeting SARS-CoV-2, the present work incorporates several distinctive features (79, 80). First, the vaccine construct was designed using immunogenic epitopes derived from both the Omicron S and N proteins, with the aim of integrating complementary humoral and cellular immune targets. Second, the construct incorporates the Gb-1 adjuvant as an immunostimulatory component and was evaluated through a comprehensive immunoinformatics workflow, including antigenicity, allergenicity, structural modeling, molecular docking, molecular dynamics-related analyses, immune simulation, and codon optimization. Collectively, these analyses provide a systematic computational assessment of the proposed vaccine candidate. Overall, our findings suggest that the proposed vaccine candidate has the potential to elicit predicted cellular and humoral immune responses against SARS-CoV-2, warranting further experimental validation.

### Study limitations and future perspectives

Despite the comprehensive computational framework employed in this study, several limitations should be acknowledged. First, epitope prediction algorithms rely on available sequence datasets, databases, and computational models, which may not fully capture the complexity of antigen processing, presentation, immunodominance, and immunogenicity under physiological conditions. Similarly, HLA population coverage analysis is based on reported allele-frequency databases and may not completely represent the genetic diversity of all global populations. Differences in database composition, prediction algorithms, and selection thresholds may therefore introduce computational bias into epitope selection and population-coverage estimates.

The 3D structure of the vaccine construct was generated through computational modeling and refinement and may not fully reflect its native conformation following recombinant expression, folding, or post-translational modifications. Likewise, molecular docking and MD simulations provide theoretical insights into potential receptor interactions and structural stability under defined computational conditions but cannot accurately reproduce the complexity of the biological environment or establish receptor activation and downstream signaling. Therefore, favorable predicted interactions between the MEV construct and immune receptors should be considered structural hypotheses rather than direct evidence of biological activity.

Furthermore, the immune responses predicted by the C-ImmSim platform represent computational simulations based on mathematical models and should not be interpreted as direct evidence of *in vivo* immunogenicity or protective immunity. Predictions of antigenicity, allergenicity, toxicity, and physicochemical properties are similarly dependent on the performance, training data, and assumptions of the computational tools employed. In addition, the present study focused primarily on selected epitopes from the S and N proteins of the Omicron variant; consequently, the construct may not encompass the full antigenic diversity of SARS-CoV-2 or account for future viral evolution and the emergence of newly circulating variants.

The contribution of the Gb-1 component to the overall immunogenicity of the proposed MEV also remains a computational design hypothesis. Although Gb-1 was incorporated based on its previously reported immunostimulatory properties, its specific contribution to the immune response of the complete MEV construct has not been experimentally established. Similarly, although the selected epitopes demonstrated favorable predicted antigenic, safety, and population-coverage characteristics, their actual processing, presentation, and recognition by immune cells require experimental confirmation.

Finally, although codon optimization and *in silico* cloning suggest the feasibility of recombinant expression, they do not guarantee efficient protein expression, correct folding, solubility, or stability in biological systems. Therefore, the proposed MEV candidate should be considered a computationally designed prototype rather than a validated vaccine. Future studies should experimentally evaluate recombinant expression and protein characterization, followed by *in vitro* immunological assays to assess antigenicity, cytokine responses, and T-cell activation. Subsequent *in vivo* studies should investigate safety, humoral and cellular immunogenicity, and protective efficacy in appropriate animal models. Ultimately, further translational and clinical investigations will be required to determine whether the predicted properties of the proposed construct translate into measurable and protective biological effects in humans.

## Conclusion

Computational immunoinformatics provides an efficient strategy for the rational design and preliminary evaluation of vaccine candidates. In this study, the SARS-CoV-2 Omicron S and N proteins were analyzed to identify predicted immunogenic epitopes for the construction of MEV. The proposed MEV candidate was assembled by linking selected linear B-cell, CTL, and HTL epitopes using GSG and GSGG linkers and incorporating the Gb-1 (GP-2-binding) peptide based on its previously reported immunostimulatory properties. Comprehensive *in silico* analyses predicted favorable physicochemical characteristics, antigenicity, and structural features, while molecular docking suggested potential interactions with selected TLRs. Collectively, these findings provide a computationally prioritized vaccine candidate and a framework for further experimental investigation. From a translational perspective, the proposed construct may serve as a starting point for subsequent vaccine-development studies aimed at evaluating its expression, stability, immunogenicity, and potential to induce protective immune responses. However, all observations reported in this study are based solely on computational predictions and should not be interpreted as experimental evidence of safety, immunogenicity, receptor activation, structural stability, or protective efficacy. Therefore, rigorous *in vitro* and *in vivo* studies are required to validate the predicted properties of the proposed MEV candidate, including recombinant production, experimental assessment of immune responses, safety evaluation, and ultimately protective efficacy. Successful experimental validation would provide the necessary basis for determining whether this computationally designed construct has further potential for translational vaccine development.

## Data Availability

The original contributions presented in the study are included in the article/supplementary material. Further inquiries can be directed to the corresponding author.
